# The PV2 cluster of parvalbumin neurons in the murine periaqueductal gray: connections and gene expression

**DOI:** 10.1007/s00429-022-02491-0

**Published:** 2022-04-29

**Authors:** Siri Leemann, Alexandre Babalian, Franck Girard, Fred Davis, Marco R. Celio

**Affiliations:** 1 Anatomy and Program in Neuroscience, Faculty of Science and Medicine, University of Fribourg, CH-1700 Fribourg, Switzerland; 2 Janelia-Farm, Howard Hughes Medical Institute, Ashburn, VA USA

**Keywords:** Orbitofrontal cortex, Hypothalamus, Su3, Autonomic nervous system, Inhibition, Allen database

## Abstract

The PV2 (Celio 1990), a cluster of parvalbumin-positive neurons located in the ventromedial region of the distal periaqueductal gray (PAG) has not been previously described as its own entity, leading us to study its extent, connections, and gene expression. It is an oval, bilateral, elongated cluster composed of approximately 475 parvalbumin-expressing neurons in a single mouse hemisphere. In its anterior portion it impinges upon the paratrochlear nucleus (Par4) and in its distal portion it is harbored in the posterodorsal raphe nucleus (PDR). It is known to receive inputs from the orbitofrontal cortex and from the parvafox nucleus in the ventrolateral hypothalamus. Using anterograde tracing methods in parvalbumin-Cre mice, the main projections of the PV2 cluster innervate the supraoculomotor periaqueductal gray (Su3) of the PAG, the parvafox nucleus of the lateral hypothalamus, the gemini nuclei of the posterior hypothalamus, the septal regions, and the diagonal band in the forebrain, as well as various nuclei within the reticular formation in the midbrain and brainstem. Within the brainstem, projections were discrete, but involved areas implicated in autonomic control. The PV2 cluster expressed various peptides and receptors, including the receptor for Adcyap1, a peptide secreted by one of its main afferences, namely, the parvafox nucleus. The expression of GAD1 and GAD2 in the region of the PV2, the presence of Vgat-1 in a subpopulation of PV2-neurons as well as the coexistence of GAD67 immunoreactivity with parvalbumin in terminal endings indicates the inhibitory nature of a subpopulation of PV2-neurons. The PV2 cluster may be part of a feedback controlling the activity of the hypothalamic parvafox and the Su3 nuclei in the periaqueductal gray.

## Introduction

The periaqueductal gray (PAG) is an extensive and complex region surrounding the cerebral aqueduct throughout the midbrain (Depaulis and Bandler 1991). The PAG became a focus of research when electrical stimulation or microinjection into different subregions of the PAG lead to antinociception and analgesia in various species (Behbehani 1995). In rodents, the PAG also plays a key role in autonomic control and behavioral responses in defense reactions (Bandler and Carrive 1988; Bandler et al. 2000, 1991; Carrive et al. 1989, 1987; Depaulis et al. 1992; Zhang et al. 1990), is implicated in cardiovascular and respiratory control (Keay et al. 1997a, 1997b; Sessle et al. 1981; Verberne and Guyenet 1992; Verberne and Struyker Boudier 1991), as well as in vocalization in rodents and primates (Yajima et al. 1980) and lordosis behavior in rats (Behbehani 1995). Thus, the PAG is assumed to be a main integrator and modulator of autonomic responses to behavioral and environmental states.

The PAG is anatomically subdivided into cyto-architectonically distinct subregions and is organized in longitudinal columns surrounding the aqueduct, namely, a dorsomedial and dorsolateral column, as well as a lateral and ventrolateral one (Bandler and Shipley 1994). Depending on whether the neurons lying lateral (or dorsolateral) to the aqueduct or those lying ventrolateral are stimulated, distinct and opposite autonomic reactions are elicited. While stimulation of the lateral PAG produces pressor responses in the cardiovascular and respiratory systems, stimulation of the ventrolateral PAG results in hypotension, bradycardia, and an overall depressor response, which includes quiescence and hyporeactivity (Depaulis et al. 1994, 1992; Keay et al. 1997b; Subramanian et al. 2008).

One of the main bidirectional connections of the PAG is with the lateral hypothalamus (Depaulis and Bandler 1991). Stimulation of the ventrolateral hypothalamus also produces pronounced autonomic effects, which include changes in blood pressure, which range from elevation to depression depending on the rostro-caudal location of the stimulation (Hess 1935; Spencer et al. 1989). Furthermore, electrical and chemical stimulation of this area elicits 50-kHz ultrasonic vocalizations (Burgdorf et al. 2007), which have been compared to the human expression of positive emotions (Panksepp and Burgdorf 2003). Within the tuberal part of the ventrolateral hypothalamus lies a population of longitudinally oriented neurons, nestled in the median forebrain bundle, between the optic tract and the fornix. This cell group–coined the parvafox nucleus (formerly PV1-Foxb1-nucleus (Alvarez-Bolado and Celio 2016)) –consists of a core of parvalbumin (Parv) immunoreactive cells, surrounded by a shell of Foxb1-positive neurons and 10% of the cells express both (Bilella et al. 2014, 2016; Meszar et al. 2012). Unlike most Parv-positive cells in the brain, the neurons within the parvafox nucleus are not GABAergic, but rather utilize glutamate as their neurotransmitter and receive a strong glycinergic input (Girard et al. 2011; Szabolcsi et al. 2017). Studies on the parvafox nucleus have revealed a possible role in vocalization (Roccaro-Waldmeyer et al. 2016; Alvarez-Bolado and Celio 2016), analgesia (Siemian et al. 2019), and body movement (Cola et al. 2020) but the precise mechanisms that are responsible for the observed effects, as well as the neural pathways involved, remain poorly understood.

Tract-tracing projection studies of the neurons in the parvafox nucleus have revealed two main terminal fields within the PAG: the supraoculomotor nucleus (Su3) and a longitudinal cluster of Parv-immunoreactive cells, initially coined the PV2 cluster, lodged within the caudal-most part of the ventromedial PAG (Bilella et al. 2016; Celio et al. 2013). We endeavored to characterize the PV2 cluster of neurons with its efferences, to determine the nature of its neurotransmitters and to search for genes expressed. Classical histological, immunofluorescence and axonal-tracing techniques were employed, completed by data mining of a gene expression database (Allen brain atlas; https://www.brain-map-org/).

## Materials and methods

### Animals

30 adult mice of both genders (17 females and 13 males), aged between 4 and 8 months and weighing 22–30 g, were used in these experiments. The anterograde tracing experiments were performed on 7 mice of the *Pvalb:Cre* genotype [129P2-Pvalb < tm1(cre)Arbr > /J], which express Cre-recombinase in Parv-expressing neurons (Hippenmeyer et al. 2005). Cre-dependent viral tracers were used to selectively target these Cre-positive cells. The study on the neurotransmitter status of the projections of the PV2 cluster were performed on 6 Slc32a-IRES-cre (VGAT-) mice and 4 Slc17a6-cre (VGlut2-) mice. C57BL/6 wild type animals were used for the topographic localization of the PV2 cluster (9 mice) and for the stereological cell quantification (6 mice). The study was conducted under the approval of the Veterinary Commission of the Canton of Fribourg [FR 2016_20] and all animals were housed in state-of-the-art animal facilities in accordance with the Swiss animal experimentation law. They were subjected to a 12 h light/dark cycle and fed ad libitum*.*

### Anterograde tracing experiments

For the analysis of the projections deriving from the PV2 cluster, 5 *Pvalb::Cre* driver mice (322/17, 324/17, 325/17, 406/15, 407/15) were injected with two different anterograde Cre-dependent adenoviral constructs, namely AAV2/1.CAG.flex.EGFP.WPRE.bGH or AAV1.CAG.flex.tdTomato.WPRE.bGH (Vector Core, University of Pennsylvania).

In preparation for the injections, the mice were anaesthetized with a mixture of Ketalar (Parke-Davis, Ann Arbor, MI; 75 mg/kg of body weight) and Xylazine (Streuli, Uznach, Switzerland; 10 mg/kg of body weight). If signs of awakening were observed, additional lower doses of anesthetic were administered. For the stereotaxic injections, the heads of the mice were secured in a special device (Kopf, model 5000) in flat-skull position and a craniotomy was performed over the target area of the brain. The adenoviral tracers were injected bilaterally in a volume of 7 nl per side or unilaterally in a volume of 7–16 nl during an interval of 0.5–1 min. The tracers were injected with a 2.5 µl Hamilton syringe via a fine-bored needle with a diameter of 0.14 mm (Ga: 34), which was connected to the stereotaxic microinjection apparatus (Kopf, model 5000). The injections into the PV2 cluster of the periaqueductal gray were made at the following coordinates: rostrocaudal Bregma: − 4.7 mm; mediolateral: ± 0.45 mm; dorsoventral: − 2.5 mm (Franklin and Paxinos 2008). The needle was left in place for an additional 3–5 min. after the injection was completed to allow for diffusion of the viral tracer throughout the injection site. The needle was then withdrawn completely, the skin above the region of the craniotomy was sutured and the animals were left to recover on a heating pad (37 °C). In post-operative care, mice received 3 × 5 µl of Buprenorphine, an opioid for pain prevention.

### Control experiments

Nine C57BL/6 wild-type mice were used for the localization of the PV2 cluster and for stereological quantification.

### Perfusion and tissue processing

After 3–4 weeks, the animals were anaesthetized with a lethal dose of pentobarbital (150–200 mg/kg body weight; Streuli, ZH). For the transcardial perfusion, the rib cage was opened, a small incision made in the right atrium of the heart and subsequently the body perfused through a fine needle placed in the left ventricle–first with a physiological (0.9%) saline solution and then with 4% paraformaldehyde (PFA) in a 0.1 M phosphate buffer (pH 7.4) at 4 °C.

The brains and spinal cords were excised, post-fixed overnight in the same solution and then transferred to a 30% sucrose solution in a 0.1 M phosphate-buffer, pH 7.3, for cryo-protection. Once the brains had sunken completely, they were cryo-sectioned into 40 or 80 µm thin sections using a cryo-mobile (Frigomobil, Reichert-Jung, Vienna, Austria). The brains were serially sliced in the coronal plane and the sections were collected in 0.1 M tris-buffered saline, pH 7.3 (TBS) containing 0.02% Sodium-azide (Na-Az). These sections were subsequently treated with antibodies against GFP (Molecular probes, Eugene, USA) to enhance the fluorescence of the terminal endings of the projections derived from the PV2 cluster of neurons. Some sections were incubated with anti-parvalbumin antibodies (Swant, Marly, Switzerland) to confirm the precision of the injection in the PV2 cluster. To determine if classical neurotransmitters were used by the PV2 cluster, antibodies against serotonin and enzyme markers for acetylcholine and catecholamine were employed (Table 2). After treatment, the sections were mounted on glass slides for histological analysis.

### Immunohistochemistry

Previously published protocols for immunofluorescence were employed (Celio 1990; Gerig and Celio 2007; Meszar et al. 2012). Sections were incubated with antibodies against GFP, Parv or various neurotransmitters, including serotonin (5-HT), the GABA-synthesizing enzyme GAD67, the enzymes tyrosine hydroxylase (TH) and choline acetyltransferase (ChAT), as well as the vesicular glutamate transporters VGlut1 and VGlut2 (Table 2). Free-floating sections collected in 24-well plates were incubated with the primary antibodies–diluted 1:400 to 1:10,000 in TBS + 0.2% Triton-X and 10% bovine serum (BSA)–at 4 °C mainly for three days. Following this primary incubation, the sections were treated either with a conjugated secondary antibody (diluted 1:200–1:500; Jackson Immuno Research, West Grove, PA) or were incubated for 2 h with a biotinylated secondary antibody (diluted 1:200, at room temperature; Vector laboratories, Burlingame, CA). This latter procedure was followed by a further exposure of 2–3 h–ikewise at room temperature and shielded from direct light exposure–to either Cy2-, Cy3- or Cy5-conjugated streptavidin (diluted 1:200, Jackson Immuno Research, West Grove, PA).

### Light microscopy, image processing and identification of the nuclear boundaries

The tissue sections were mounted, cover-slipped and analyzed in either a Leica 6000 epifluorescence microscope (equipped with a Hamamatsu C4742-95 camera), a digital slide-scanner (Nanozoomer, Hamamatsu), or a Leica TCS SP5 confocal laser microscope. The images were post-processed for brightness and contrast in Adobe Photoshop and image stacks were produced with ImageJ2. The figures were assembled in Adobe Illustrator CC. Nuclear boundaries were not delineated using Nissl-stained sections as a reference, but by comparing the images to those of the mouse atlas (Franklin and Paxinos 2008).

### Stereological quantification (Table 3)

The neurons of the PV2 cluster were counted using stereological techniques. Six C57BL/6 wild type animals were deeply anaesthetized, perfused, fixed and their brains excised, as previously described. The free-floating sections were incubated with an antibody against parvalbumin (PV27, Swant Inc, Marly, Switzerland) and the region of the cluster was determined with an epifluorescence microscope (Leica 6000). The selected sections were then mounted and cover-slipped for quantification. Quantification was performed using the Optical Fractionator Workflow in the Stereo-Investigator 11.09 (MBF Bioscience, Williston, VT). Counts were made on uniform random systematic samples of every second coronal section. For the estimations, contours were drawn around the region of interest. Counting frames of 110 × 80 µm were placed at 150 µm intervals along the x- and y-axes. Tissue thickness was measured for each animal at an average of 18 µm. Additionally, tissue thickness was re-evaluated at every fifth counting site. The grid size was selected to attain a reasonable coefficient of error (CE). The CE indicates the precision of the estimation and a CE of below 0.15 was determined as acceptable for these experiments (Table 3).

### Gene expression analysis

A gene expression analysis was performed to generate a list of gene encoding proteins, ion channels or receptors, with enriched expression in the region of the PV2 cluster. The list of gene expressed in the PV2 cluster was then compared to the gene expressed in Parv neurons of the brain (reticular thalamic nucleus) or in the parvafox nucleus, a hypothalamic nucleus innervating the PV2 cluster.

To identify genes that are locally enriched in the region of the PV2 cluster, the Allen Database was screened using the Anatomic Gene Expression Atlas (AGEA) tool, an interactive atlas based on *in-situ* hybridization gene expression data in the Allen Mouse Brain Atlas (https://www.brain-map.org/). With the gene finder tool, a list of genes was generated and revealed genes with enhanced expression in the defined voxel. In the case of the PV2 cluster the examined voxels included the localizations: 10,000/4000/6000; 10,200/4000/6000 and 10,200/4000/6200. These lists were then screened online for genes enriched specifically in the region of the PV2 cluster (Table 4). To determine the genes whose expression is similar to the expression of Parv in the region of the periaqueductal gray encompassing the PV2 cluster, an Allenminer similarity search was performed (Davis and Eddy 2009). This list was then screened online for genes that were restricted to the area corresponding to the PV2 cluster (Table 4). Additionally, the results were compared to previous gene expression analyses, which included the region of the parvafox nucleus and the reticular thalamic nucleus (Girard et al. 2011; Szabolcsi et al. 2017), a region enriched with Parv expressing neurons (Table 4).

## Results

**Fig. 1:**
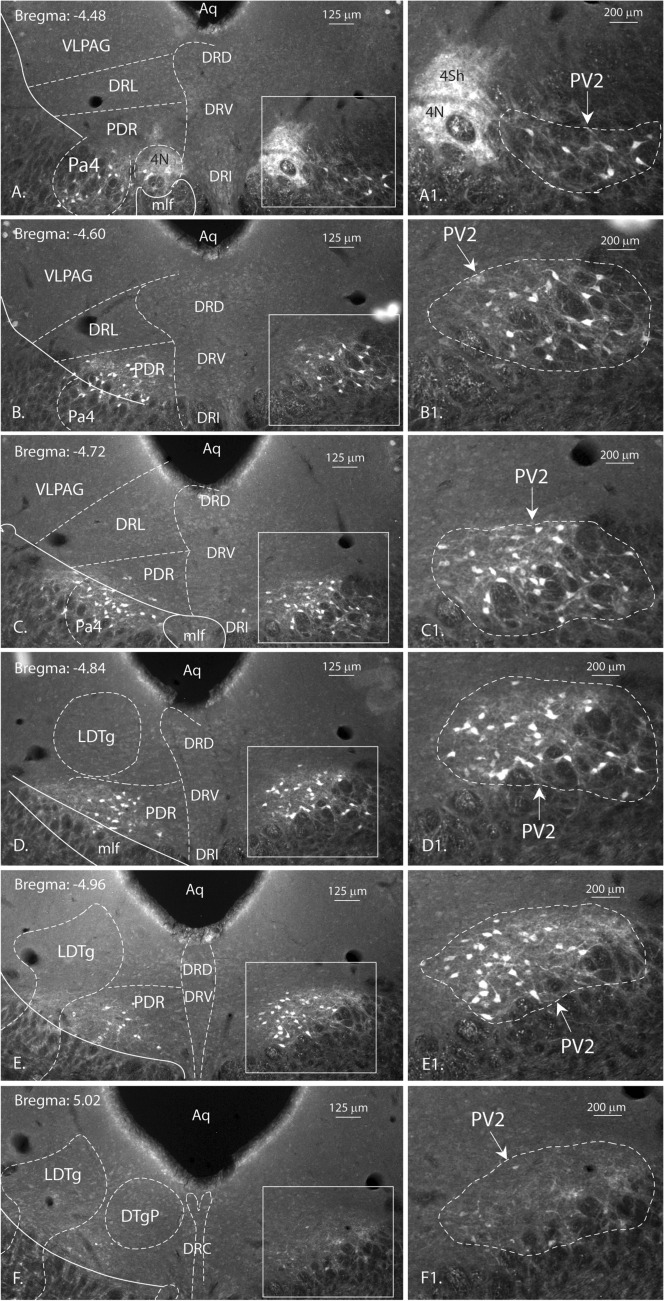
Localization of the Parv expressing neurons of the PV2 cluster. Six consecutive epifluorescent images of the bilateral PV2 cluster localization from rostral (**A** Bregma: − 4.48) to caudal (**F** Bregma: − 5.02) in the most caudal part of the periaqueductal gray. The boxed portions correspond to the higher magnification images on the right (A1–F1). The location of Parv-positive neurons in the paratrochlear nucleus (Pa4) and in the posterodorsal raphe nucleus (PDR) are visible in images **A**, **B**, **C**, respectively **B**, **C**, **D** and **E**. The proximity of PV2 cluster neurons to known structures like the trochlear nucleus (4 N) and the laterodorsal tegmental nucleus (LDTg) are shown in **A**, respectively in **E** and **F**. *Aqueductus cerebri* (Aq); dorsal raphe nucleus, caudal part (DRC); dorsal raphe nucleus, dorsal part (DRD); dorsal raphe nucleus, interfascicular part (DRI); dorsal raphe nucleus, ventral part (DRV); dorsal raphe nucleus, caudal part (DRC); dorsal tegmental nucleus, pericentral part (DTgP); medial longitudinal fascicle (mlf); shell region of the trochlear nucleus (4Sh); ventrolateral region of the periaqueductal gray (VLPAG)

The PV2 cluster of the caudal murine periaqueductal gray is a bilateral, elongated, clearly delineated group of Parv immunopositive cells (Fig. 1). This cluster of cells is located ventrolateral to the aqueduct, lateral to the dorsal raphe nucleus and medioventral to the ventrolateral column of the PAG described by Bandler (Bandler and Shipley 1994). It overlaps partially with the paratrochlear nucleus (Pa4) in its rostral portion and with the posterodorsal raphe and the laterodorsal tegmental nuclei in its caudal part (Fig. 1, right column). It intrudes the reticular formation just beside the PAG, while spanning over the bregma levels − 4.56 and − 4.96. Its dorsoventral and mediolateral coordinates are − 2.5 and ± 0.45, respectively. Generally, 15–20 cells are visible on each coronal section, with sections containing up to 30 or more. The Parv-immunopositive cells of the cluster are mostly small to medium sized bi- or multipolar neurons. Some of the neurons possess large and thick axons. The smaller neurons showed a slightly weaker immunoreactivity. Tract-tracing studies were performed to determine the projections of the PV2-axons.

### Efferent projections of the PV2 cluster: anterograde tracing experiments (Table 1 and Fig. 2)

The most precise injections of the PV2 cluster were found in cases 325/17, 406/15 and 407/15 and a list of the structures containing terminals in all three cases is provided in Table 1.

**Table 1:** List of efferences from the PV2- cluster found in 3 different injections (325-17; 406-15 and 407-15)

Brain area	Region	Abbreviation	Projections from PV2		
			325/17	406/15	407/15
Telencephalon					
	Nucleus of the horizontal limb of the diagonal band	HDB	+	+ + +	+ +
	Lateral septal nucleus, intermediate part	LSI	+	+	+
	Medial septal nucleus	MS	+	+ +	+ +
	Piriform cortex	Pir	+	+	+
	Nucleus of the vertical limb of the diagonal band	VDB	+	+ + +	+
Diencephalon					
	Central medial thalamic nucleus	CM	+ +	+	+
	Paracentral thalamic nucleus	PC	+ +	+	+
	Parafascicular thalamic nucleus	PF	+ + +	+	+
	Gemini hypothalamic nucleus	Gem	+ +	+ + +	+ + +
	Parvafox nucleus	parvafox	+ +	+ + +	+ +
Mesencephalon					
	Medial parabrachial nucleus	MPB	+ +	+ +	+ +
	Mesencephalic reticular formation	mRt	+ + +	+ +	+ +
	Parabrachial pigmented nucleus of the VTA	PBP	+ +	+	+
	Posterodorsal raphe nucleus	PDR	+ + +	+ +	+ +
	Parvalbumin nucleus 2	PV2	Injection	Injection	Injection
	Supraoculomotor nucleus	Su3	+ + +	+ +	+ + +
	Dorsomedial tegmental area	DMTg	+ +	+	+ +
	Dorsal tegmental nucleus central part	DTgC	+	+ +	+ +
	Laterodorsal tegmental nucleus	LDTg	+ + +	+ + +	+
	Laterodorsal tegmental nucleus, ventral part	LDTgV	+ + +	+	+ +
	Pontine reticular nucleus caudal part	PnC	+ +	+ +	+
	Pontine reticular nucleus oral part	PnO	+ +	+ +	+ +
	Reticulotegmental nucleus of the pons	RtTg	+ + +	+ +	+ +
	Ventral tegmental area	VTA	+ + +	+ +	+ +
	Intermediate gray layer of the superior colliculus	InG	+ +	+ +	+
	Intermediate white layer of the superior colliculus	InWh	+ +	+ +	+
Rhombencephalon	Ambiguus nucleus	Amb	+	+	+
	Botzinger complex	Bo	+	+	+
	Gigantocellular reticular nucleus	Gi	+ + +	+ +	+
	Intermediate reticular nucleus	Irt	+ +	+ +	+ +
	Raphe interpositius nucleus	RIP	+	+	+
	Rostroventral reticular nucleus	RVL	+	+	+
	Solitary nucleus, ventral part	SolV	+	+	+

The number of + symbols reflect the density of the projections: + : low; +  + : strong; +  +  + : very strong

**Fig. 2:**
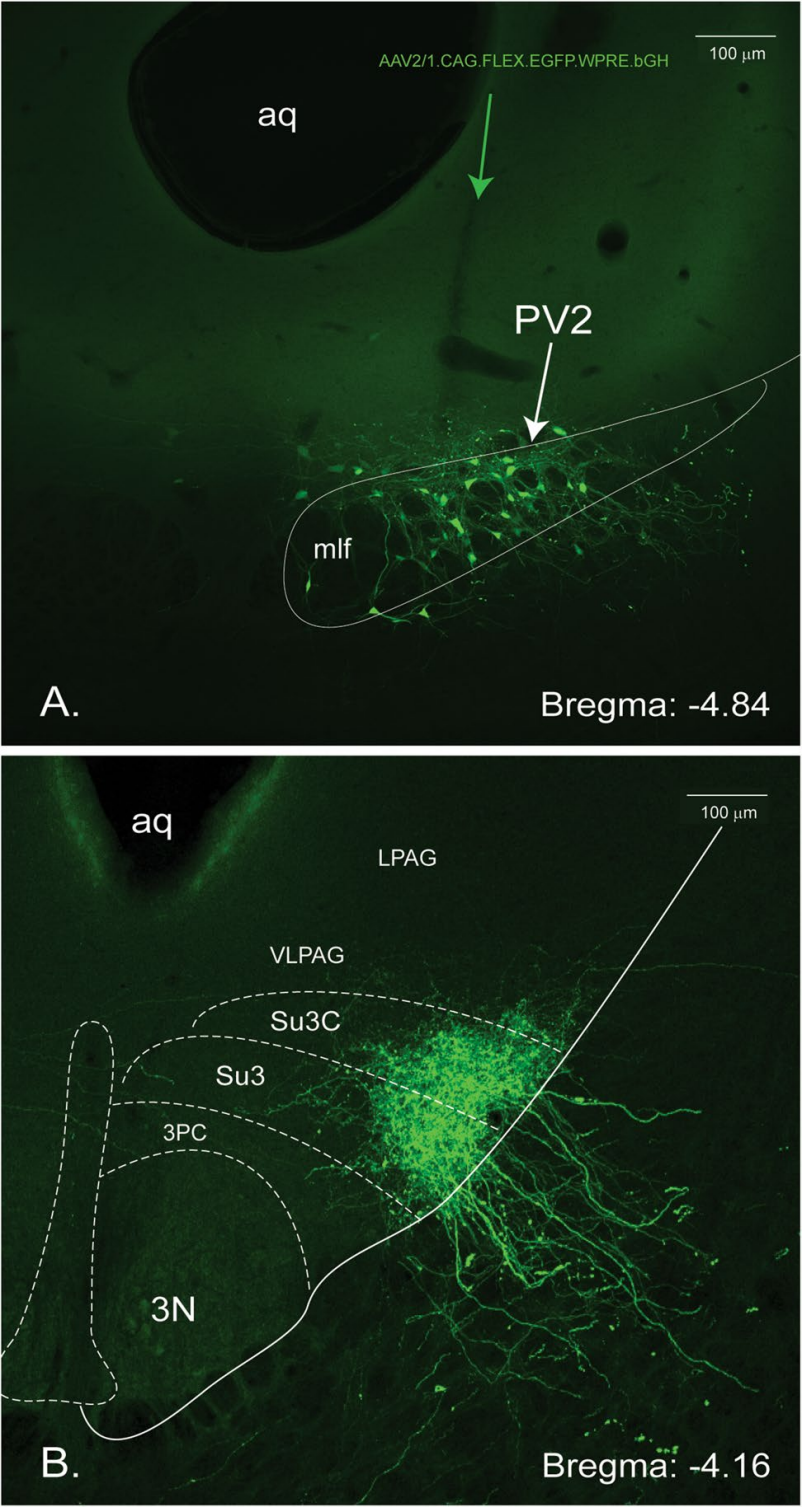
Localization of the injection site of the EGFP-fluorescent AAV-tracer in the midbrain and of the terminals in the Su3 region (brain 325-17). **A** The cell bodies of the PV2 cluster, scattered between the axons of the medial longitudinal fascicle, have been infected with the anterograde, Cre-dependent tracer (green). The trajectory of the injection syringe is indicated with the green arrow. **B** Dense innervation of the Su3-nucleus and its cap (Su3C) with terminals of axons originating in the PV2 cluster (GFP). *Aq* aqueductus cerebri, 3N oculomotor nucleus, *3PC* oculomotor nucleus, parvicellular part, *VLPAG* ventrolateral periaqueductal gray. *LPAG* lateral periaqueductal gray. *Su3* supraoculomotor PAG, *Su3C* supraoculomotor cap

The PV2 cluster projects to all parts of the brain, from the telencephalon to the myelencephalon. In the spinal cord, virtually no label was found. A few structures received more dense projections than others and this is reflected by the number of + symbols in Table 1. While most of the projections were only found ipsilaterally, some were also observed as a mirror image on the contralateral half (e.g., the contralateral parvafox nucleus, Fig. 4a and the contralateral PV2 cluster). The strongest projections will be described systematically from rostral to caudal in the following paragraphs and are listed in Table 1. Figure 2A provides a representative injection site in the PV2 cluster of mouse 325-17, illustrating the Parv-cre neurons and their entwined dendrites infected with AAV tracer (Fig. 2A, green). One of the strongest projection terminates in the Su3- and Su3c nuclei (Fig. 2B).

### Telencephalic projections (Fig. 3)

**Fig. 3:**
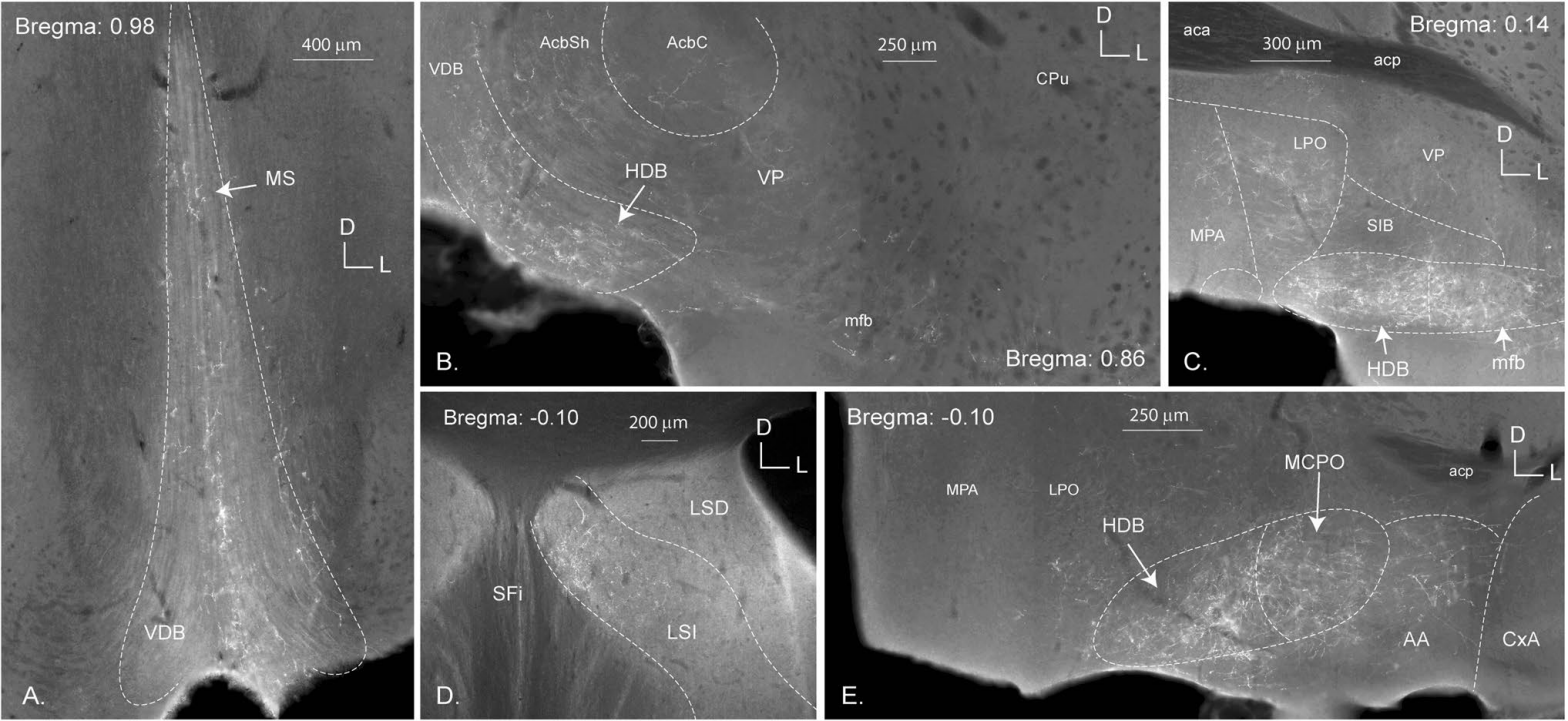
Telencephalic projections of the PV2 cluster. Images of coronal sections of the forebrain projections from the PV2 cluster from proximal (**A** Bregma: 0.98) to distal (**E** Bregma: − 0.10). The PV2 cluster is shown to project to the medial septum (MS; **A**), the vertical limb of the nucleus of the diagonal band (VDB; **A**) and the intermediate part of the lateral septum (LSI; **D**). High-density fibers are found in the horizontal limb of the nucleus of the diagonal band (HDB; **B**, **C** and **E**). Terminals are also seen in the lateral (LPO; **C**), the medial (MPA; **C**) and the magnocellular (MCPO; **E**) preoptic areas, as well as in the medial forebrain bundle (mfb; **C**) and the anterior amygdala (AA; **E**). D-L: dorso-lateral axes

The ascending fibers most likely followed the medial forebrain bundle (*mfb)* through the diencephalon to the telencephalon. The fibers crossed the preoptic areas, as these showed light staining in two of the three cases. In all specimens, terminals were observed in the diagonal band and the nuclei of both the horizontal and vertical limbs. These fibers continued rostrally to the medial and lateral septal nuclei (Fig. 3). Finally, the piriform cortex received sparse projections as well.

### Diencephalic projections (Fig. 4)

**Fig. 4:**
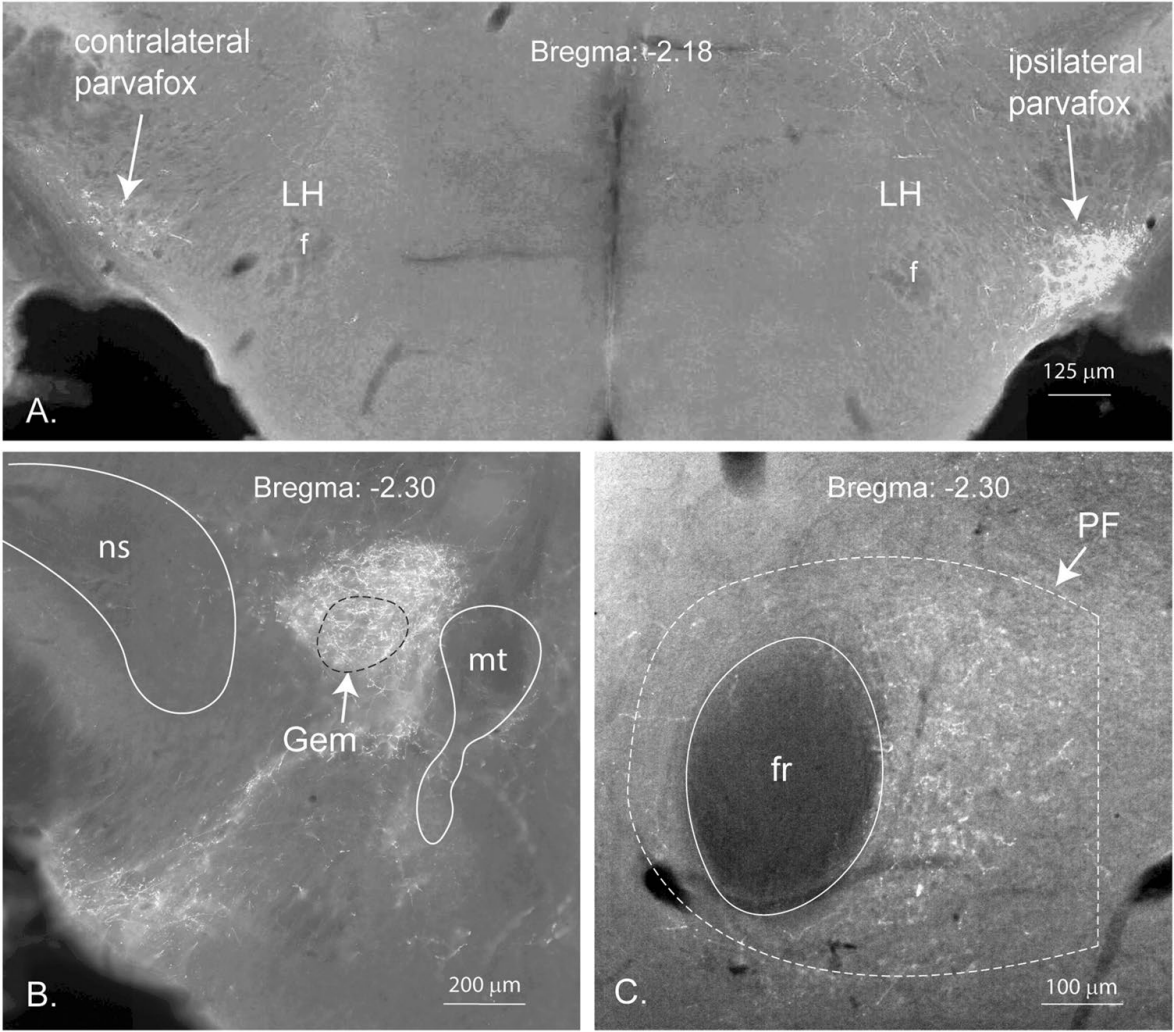
Diencephalic projections of the PV2 cluster (brain 325-17). **A** Bilateral projections to the parvafox nucleus of the lateral hypothalamus (LH); the stronger labelling is on the ipsilateral parvafox nucleus (right side of the picture); *f* fornix. **B** Image of terminals in and around the gemini nucleus (Gem). *mt* mammillo-thalamic tract; *ns* nigro-striatal tract. **C** Terminals located around the fasciculus retroflexus (fr) within the thalamic parafascicular nucleus (PF)

In the hypothalamus, the axons stemming from the PV2 cluster terminated mainly in two structures, namely the parvafox (Fig. 4A) and the gemini nuclei (Fig. 4B). The axon terminals in the region of the gemini nuclei occupied a larger surface than the boundaries depicted in the atlas which was used as a reference (Franklin and Paxinos 2008). According to the reference atlas, the terminals extended beyond the borders of the Gemini nucleus caudally into the ventral tegmental area. In two cases, the lateral hypothalamus received terminals in an area larger than the parvafox nucleus.

In the thalamus, some fibers terminated in the central and paracentral nuclei, but the structure containing the largest number of terminals was the more caudal parafascicular thalamic nucleus (Fig. 4C).

### Mesencephalic projections (Fig. 5)

**Fig. 5:**
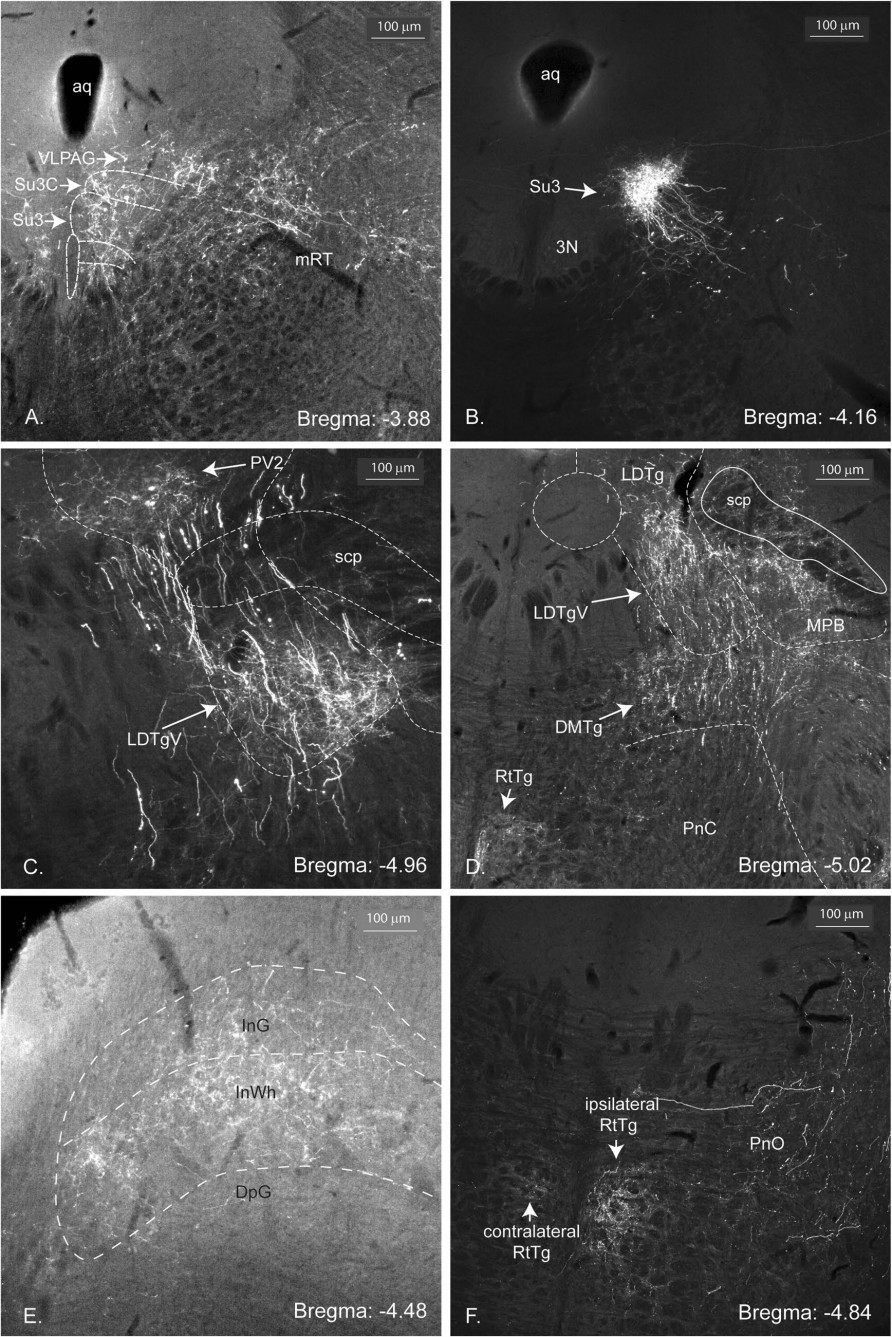
Midbrain projections of the PV2 cluster (brain 325-17). Epifluorescent images of the projections from the PV2 cluster to the midbrain. **A** Fibres densely innervate the supraoculomotor nucleus (Su3) and its cap (Su3C). Terminals are also seen in the ventrolateral periaqueductal gray (VLPAG) and in the mesencephalic reticular formation (mRT). **B** More caudal image showing the dense innervation of the Su3-nucleus. **C** At the caudal end of the PV2 cluster, fibres and terminals are present in the ventral part of the laterodorsal tegmental nucleus (LDTgV). **D** The terminals in the LDTgV continue caudally and are found also in the medial parabrachial nucleus (MPB). The dorsomedial tegmental nucleus (DMTg) and the reticulotegmental nucleus (RtTg; also, in F) also receive terminals. **E** The intermediate gray and white layers of the superior colliculus also receive projections. **F** The oral part of the pontine reticular formation shows terminals around the RtTg, stronger on the ipsi- than on the contralateral side

The most striking terminal field of the midbrain was in the supraoculomotor nucleus (Su3) of the PAG, both ipsi- (Figs. 2B, 5B) and contralaterally (not shown). Surrounding the PAG, the mesencephalic reticular formation, the posterodorsal raphe nucleus and the parabrachial pigmented nucleus contained terminals.

In the tegmentum, fibers terminated densely in the dorsomedial and laterodorsal tegmental nuclei (Fig. 5C, D), in the medial parabrachial nucleus as well as in the pontine reticular nucleus, ventral tegmental area and reticulotegmental nucleus (RtTg; Figs. 5D, F). Furthermore, the contralateral laterodorsal tegmental nucleus also received terminals. The intermediate layers of the superior colliculus were innervated in all three cases.

### Metencephalic projections (Fig. 6)

**Fig. 6:**
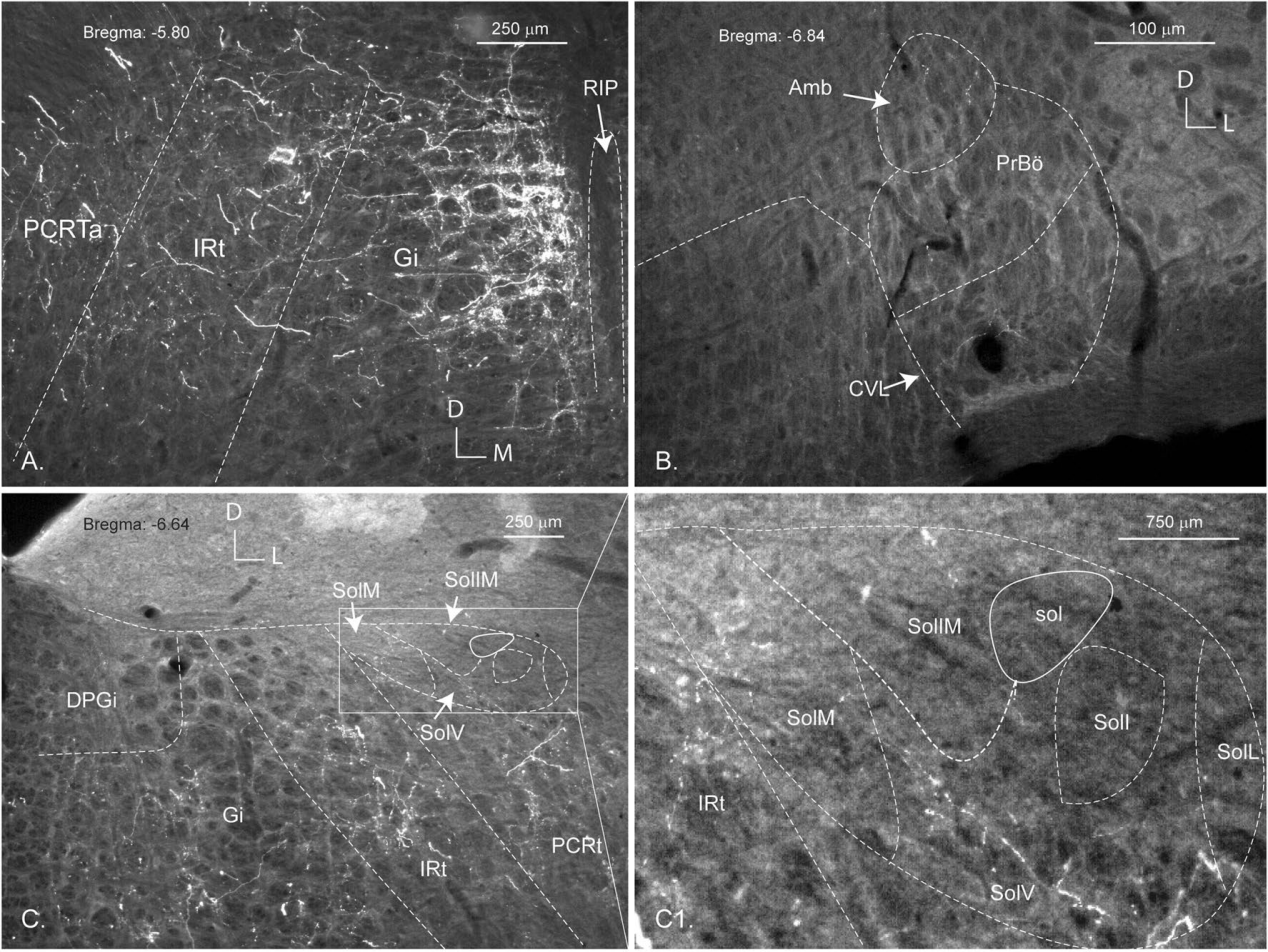
Epifluorescent images of the hindbrain projections from the PV2 cluster (brain 325-17). The gigantocellular nucleus (Gi) and intermediate reticular formation (IRt) are densely innervated. The alpha part of the parvocellular reticular nucleus (PCRTa) also receives projections (**A**, **C**). Terminals are seen in the medial (SolM) and ventral (SolV) part of the nucleus of the solitary tract (NTS; **C**; higher magnification image in C1). Only sparse labelling is seen in the *Nucleus ambiguus* (Amb), the pre-Bötzinger complex (PrBö) and the caudal ventrolateral medulla (CVL; all in **B**), as well as in the intermediate part of the NTS (**C** and C1). D-M and D-L: dorso-medial and dorso-lateral axes

The strongest projections terminated in the gigantocellular and intermediate reticular nuclei (Fig. 6A). Further structures, which received projections in all three cases, include the *Nucleus ambiguus*, the Bötzinger complex, the raphe interpositus nucleus and the rostroventral reticular nucleus (Fig. 6B). Fibers also terminated in the solitary nucleus in all three cases, where most of the terminals were found in its ventral part (Fig. 6C, C1).

### Neuronal chemical identity (Table 2)

**Fig. 7:**
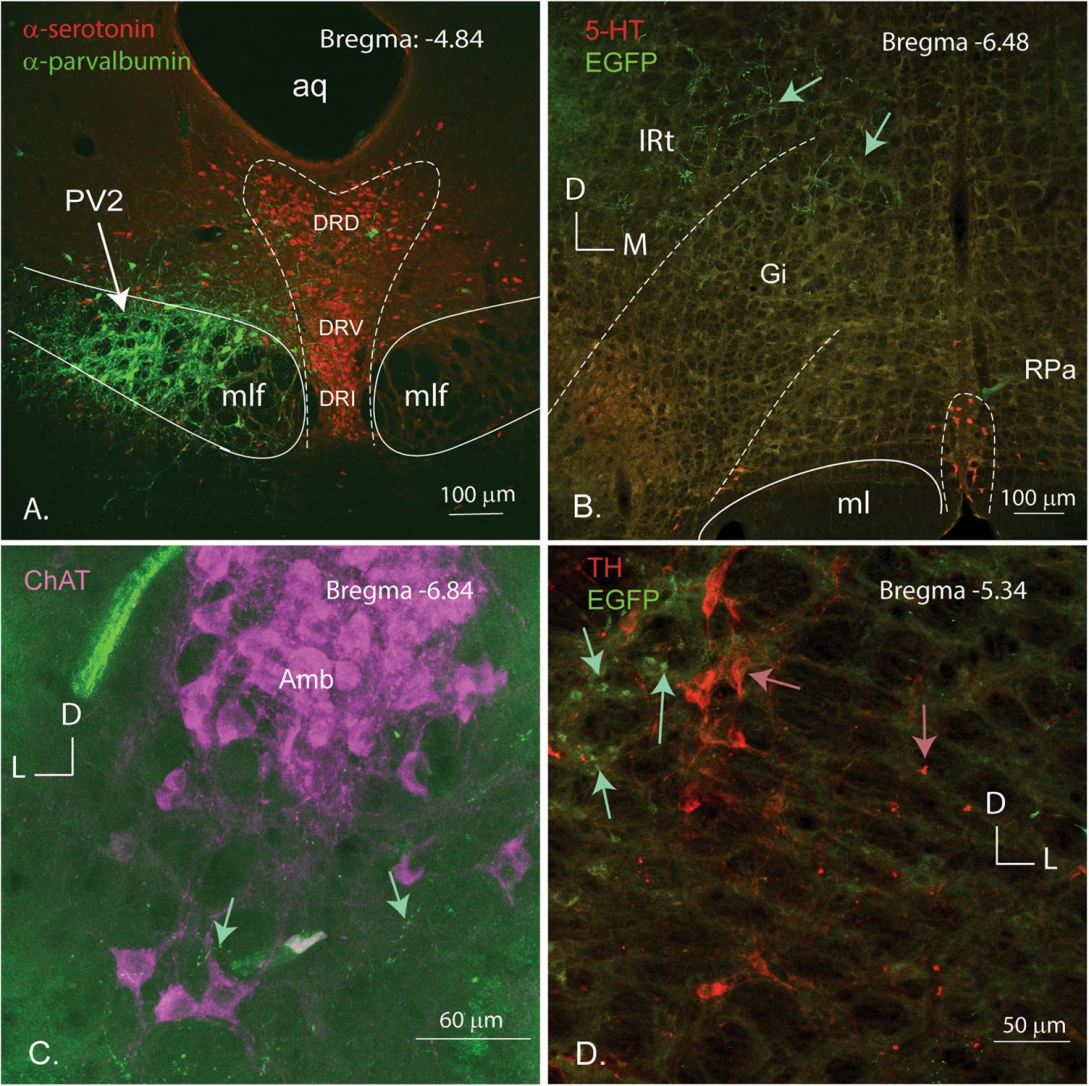
Neurotransmitters or enzymes indicating that catecholamines or acetylcholine are not related to the neurons of the PV2 cluster. **A** Immunostaining of the PV2 cluster in the medial longitudinal fasciculus with anti-Parv (green) and its relations to the serotonergic dorsal, ventral and interfascicular parts of the dorsal raphe nucleus (DRD, DRV and DRI; red) (brain 407-15). Confocal images of regions within the brainstem showing that there is no co-localization between the projections of Parv neurons of the PV2 cluster (green arrows) and the neurotransmitters serotonin (**B**, brain 407-15), and the enzymes choline acetyltransferase (**C**) tyrosine hydroxylase (**D**) (red arrows). mlf: medial longitudinal fascicle; Amb: *Nucleus ambiguus*; L-D and D-L: laterodorsal and dorsolateral axes

Coronal sections of the brain of *Pavalb::Cre* mice were incubated with antibodies against various neurotransmitters including serotonin (5-HT) and the precursor enzymes tyrosine hydroxylase (TH) and choline acetyltransferase (ChAT), markers for the presence of catecholamines and acetylcholine, respectively (Fig. 7). No evidence of immunoreactivity to any of these neurotransmitters was observed in the PV2 cluster and no co-labelling was remarked with the above markers in any of the terminal ending regions. As Fig. 7A shows, the immunostaining for serotonin allows us to demonstrate that the PV2 cluster lies neither in the dorsal raphe nor exclusively in the posterior dorsal raphe nuclei, but rather forms its own entity (Table 2).

**Table 2:** Primary and secondary antibodies and avidins used for localizing various antigens by immunofluorescence

Primary antibody	Host	Antibody type	Dilution factor	Manufacturer	Catalog no	Lot no
ChAT	Rabbit	Monoclonal IgG	1:1000—5000	Abcam, Cambridge, UK	ab178850	EPR16590
GAD67	Mouse	Monoclonal IgG	1:1000	Millipore	MAB5406	
GFP	Rabbit	Polyclonal	1:2000	Molecular Probes, Eugene, USA	A6455	1,650,113
GFP	Chicken	Polyclonal	1:400	AvES labs inc., Tigard, USA	GFP-1020	GFP697986
PV27	Rabbit	Polyclonal IgG	1:3000	Swant, Inc., Marly, Switzerland		
PV235	Mouse	Monoclonal IgG	1:1000—5000	Swant, Inc., Marly, Switzerland		10-11F
Serotonin (5-HT)	Rabbit	Polyclonal	1:1000	Immuno nuclear corporation, Stillwater, USA	43H2TR	8,344,016
Tyrosine hydroxylase (TH)	Mouse	Monoclonal IgG	1:1000—3,000	Immunostar, Hudson, USA	22,941	1,602,001
VGLut1	Rabbit	Polyclonal	1:1000—3000	Synaptic systems	135,403	AB_887883
VGlut2	Rabbit	Polyclonal	1:1000—30000	Synaptic systems	135,403	AB_2315570
*Biotinylated secondary antibody*						
Biotinylated anti-mouse	Horse	IgG (H + L)	1:250	Vector laboratories, Burlingame, Ca, USA	BA-2000	ZA0409/Z0715
Biotinylated anti-rabbit	Goat	IgG (H + L)	1:250	Vector laboratories, Burlingame, Ca, USA	BA-1000	ZB1007
*Fluorochrome-conjugated secondary antibody*						
Alexa fluor 488 anti-mouse	Donkey	IgG (H + L)	1:200	Jackson immuno research, Suffolk, UK	A21202	1,226,927
Alexa fluor™ 488 anti-rabbit	Donkey	IgG (H + L)	1:200	Jackson immuno research, Suffolk, UK	A21206	1,834,802
Alexa fluor 488 anti-chicken	Donkey	IgG (H + L)	1:200	Jackson immuno research, Suffolk, UK	703-225-155	
Cy3 anti-chicken	Donkey	IgG (H + L)	1:200	Jackson immuno research, Suffolk, UK	703-165-155	120,431
Cy3 anti-mouse	Donkey	IgG (H + L)	1:200	Jackson immuno research, Suffolk, UK		
Cy5 anti-rabbit	Donkey	IgG (H + L)	1:200	Jackson immuno research, Suffolk, UK		
*Antibody detection*						
Streptavidine Cy2			1:200	Jackson immuno research, Suffolk, UK	016-540-084	
Streptavidine Cy3			1:200	Jackson immuno research, Suffolk, UK	016-160-084	

**Fig. 8:**
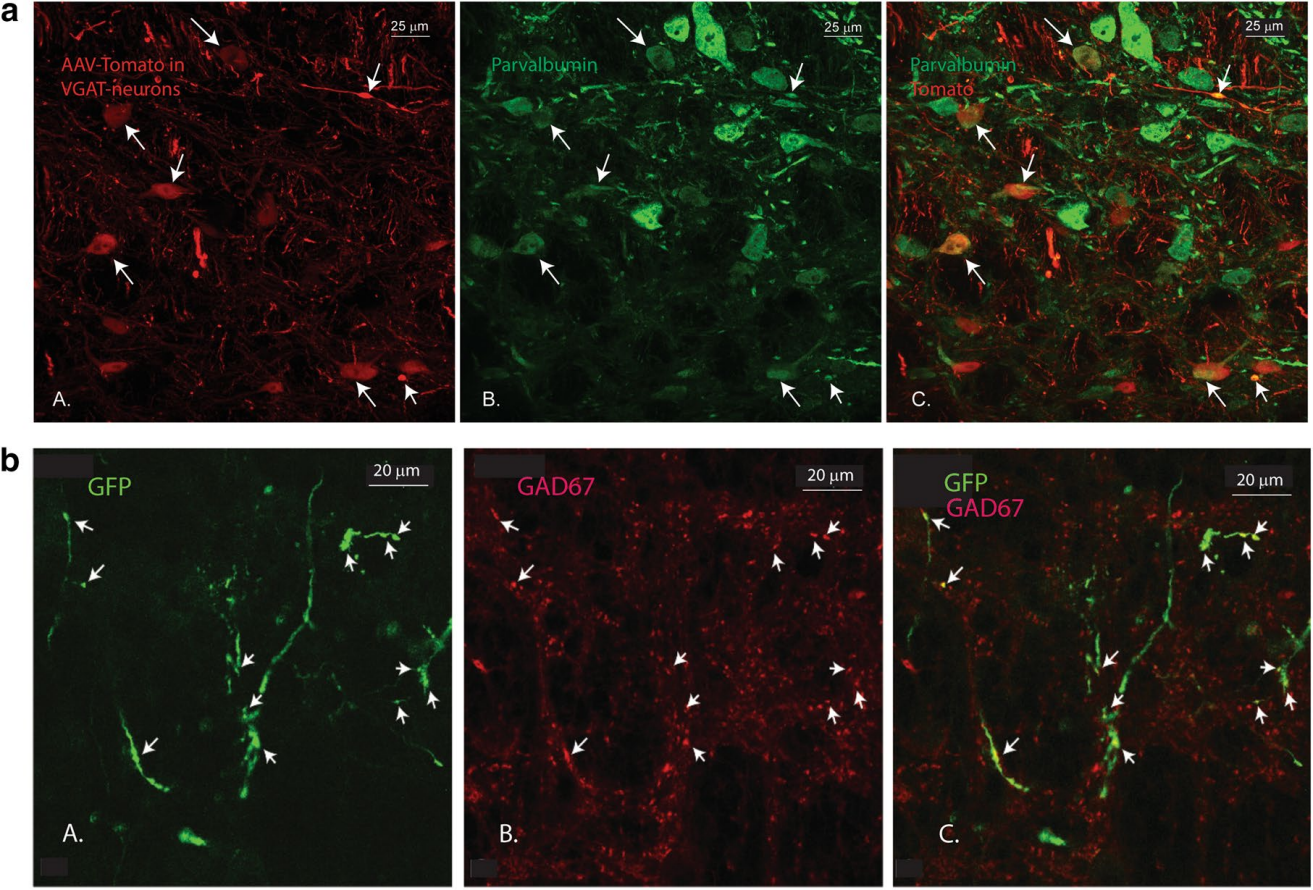
**A** Co-localization between markers for GABA transmission and Parv in the PV2 cluster. Confocal images from a VGAT-Cre mouse brain, showing in red the neurons of the PV2 cluster infected with the Cre-dependent, tomato labelled viral tracer (panel **A**) and in green the Parv-positive neurons (panel **B**). Panel **C** shows the merging of the two colors and the arrows point to the neurons which show a co-localization (yellow). Panel **B** Co-localization between GAD67 and the terminals of the GFP-axon projections from the PV2 cluster. Confocal images illustrating the terminal endings from the PV2 cluster (green) (panel **A**) and the distribution of GAD67 (red) in the GABAergic terminals (panel **B**) in the region of the gigantocellular nucleus (Gi). The arrows in panel C indicate the merging of the red and green fluorescence’s to a yellow colour, indicating co-localization

A subpopulation of neurons of the PV2 cluster and its terminals express GABA-markers, thus suggesting that they are inhibitory. The injection of a Cre-dependent tomato tracer in the PV2 cluster of a VGAT-cre mice (Fig. 8A), led to a co-localization of GABA (in red) in some of the Parv positive neurons (in green). Figure 8B shows the co-labelling of terminals derived from the PV2 cluster (green) and GAD67-immunofluorescence (red) in a region of the gigantocellular reticular nucleus of the hindbrain. In contrast, no co-localization was visible in the VGlut2-cre mice that were injected with the same Cre-dependent tracer.

### Cell counting in the PV2 cluster (Table 3)

Once immunostaining with anti-Parv antibodies was performed on the cryo-sectioned brains of C57Bl/6 wildtype mice (*n* = 6, both female and male) to locate the PV2 cluster, stereological quantification using the optical fractionator was performed. Cells were counted bilaterally and evaluated accordingly. The number of Parv-immunoreactive cells in the PV2 cluster was 507 ± 127 on the right side and 442 ± 56 on the left. An overall mean of 475 cells per cluster per hemisphere was observed. Neither significant right-left differences nor sexual dimorphisms were observed (Table 3).

**Table 3:** Stereological quantification of Parv-positive neurons in the PV2 cluster

Case Nr	Side	Marker	Region	Estimated population (using mean section thickness)	CE
142-18	Left	PV	PV2	359.83	0.12
142-18	Right	PV	PV2	487.72	0.09
143-18	Left	PV	PV2	465.48	0.1
143-18	Right	PV	PV2	423.14	0.08
144-18	Left	PV	PV2	484.81	0.09
144-18	Right	PV	PV2	613.4	0.09
145-18	Left	PV	PV2	442.33	0.08
145-18	Right	PV	PV2	500.27	0.08
146-18	Left	PV	PV2	394.43	0.1
146-18	Right	PV	PV2	331.22	0.15
147-18	Left	PV	PV2	505.32	0.09
147-18	Right	PV	PV2	684.76	0.07
Mean overall				474.39	
Mean L				442.03	
Mean R				506.7516667	
SD overall				99.6544301	
SD L				55.51278219	
SD R				127.4890794	

### Gene expression analysis (Table 4)

**Fig. 9:**
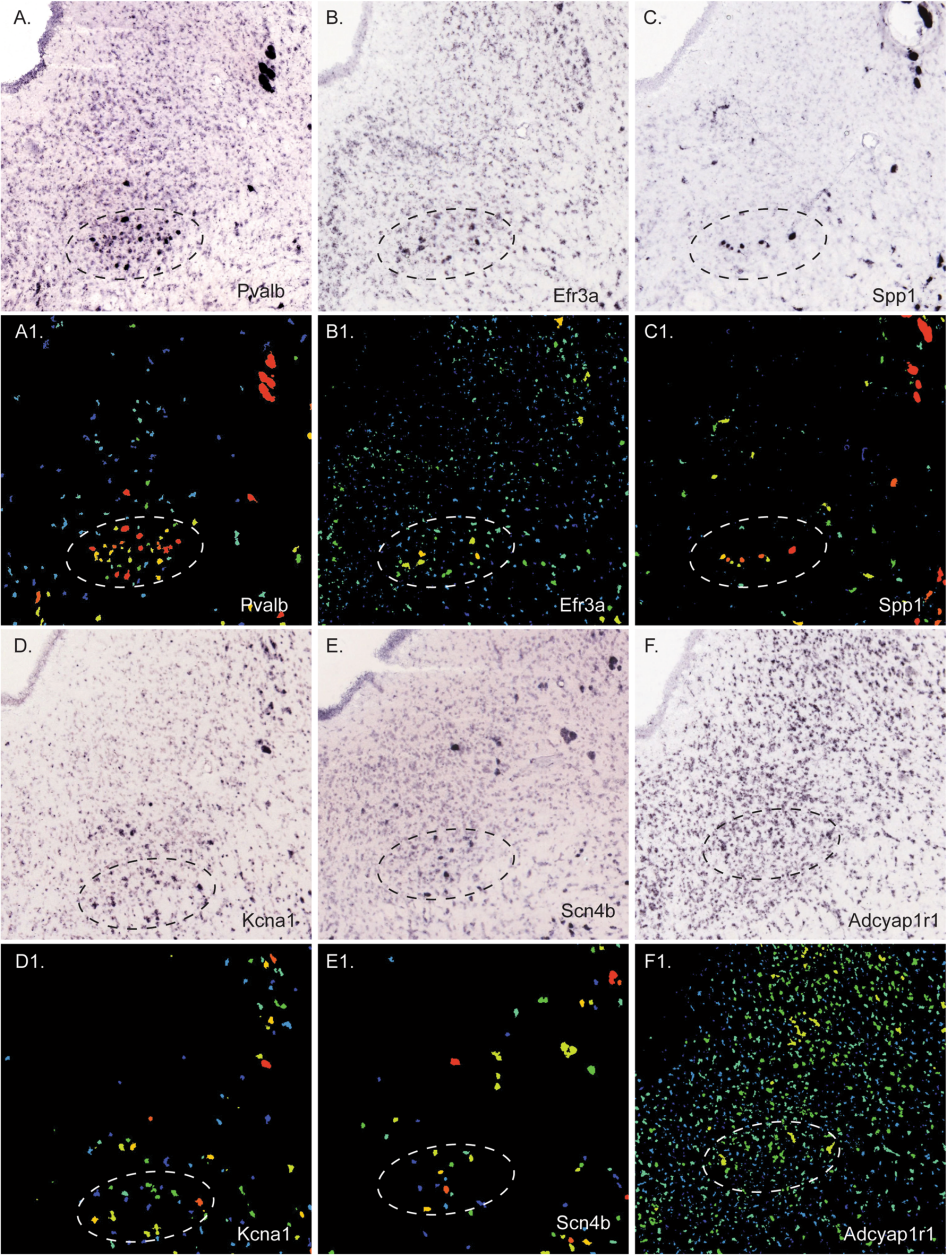
In situ hybridization (ISH) images of representative genes expressed in the PV2 cluster. Representative ISH images and the corresponding expression of *Parv* (**A**, A1), *Efr3a* (**B**, B1), *Spp1* (**C**, C1): *Kcna1* (**D**, D1), *Scn4b* (**E**, E1): and *Adcyap1r1* (**F**, F1). In addition to the standard ISH image, the Allen Database offers an expression image, in which the strength of the ISH reaction is quantified: the red color indicates strong expression, yellow indicates intermediate expression and green indicate low expression. The oval shows the approximate location of the PV2 cluster. All images are downloaded from the Allen Brain Atlas database (http://mouse.brain-map.org)

Table 4 contains a detailed and complete list of the genes expressed preferentially in the PV2 cluster of the murine PAG. This list includes genes encoding proteins that can be classified as follows: ion channel activity: potassium (*Hcn2*, *Kcna1*, *Kcna2*, *Kcnab2*, *Kcnab3*, *Kcnc1*, *Kcnc3*, *Kcnk1*) and sodium: (*Scn4b*), transporter activity (*Slc17a6*, *Slc39a14*, *Vamp1*), enzyme activity (*Cpn2*, *Ccni*, *Emb*, *Gad1*, *Gad2*, *Inpp5j*, *Serpini1*), receptor activity (*Adcyap1r1*, *Chrm2*, *Crhr1*, *Glra1*), receptor inhibitor activity (*Lynx1*), neuropeptide/neurohormone activity (*Adcyap1*, *Nxph4*, *Penk*, *Pnoc*), cytoskeletal structural components (*Nefh*), extracellular matrix/cell adhesion molecules (*Cygb*, *Spp1*, *Emb*, *Spp1*) and genes of unclear function (*Efr3a*, *Sncg*, *IQseq3*). Figure 9 displays representative images of the *in-situ* hybridization images taken from the Allen Brain Atlas database of the respective genes in this region compared to the expression of *Pvalb*. Some of the genes (e.g., *Spp1*, *Efr3a*, *Kcna1*) showed a very faint but almost exclusive expression in the PV2 cluster in and around the PAG, whereas others (e.g., *Adcyap1r1*, *Gad1*, *Gad2*) were expressed more strongly but throughout the entire lateral and/or ventrolateral PAG and LDTg. Comparisons were made to previous studies of the parvafox nucleus (Girard et al. 2011; Szabolcsi et al. 2017) and the reticular nucleus of the thalamus (data gained from the ABA). The genes that are expressed in these regions are remarked in the last three columns of Table 4. The genes that are expressed in both the PV2 cluster and the parvafox nuclei, but not in the reticular nucleus include: *Adcyap1*, *Glra1*, *Kcna1*, *Lynx1*, *Nxph4*, *Penk* and *Slc17a6*. Genes expressed in all three regions include those mentioned above and additionally: *Crhr1*, *Efr3a*, *Hcn2*, various other genes encoding potassium channels (*Kcna2*, *Kcnab2*, *Kcnab3*, *Kcnc1*, *Kcnk1*), *Nefh*, *Penk*, *Scn4b*, *Serpini1*, *Spp1* and *Vamp1*. The last column contains the genes that were also found in the reticular thalamic nucleus, suggesting that some of these are ubiquitously expressed in Parv-positive cells. Finally, some genes were found to be expressed in only the reticular thalamus and the PV2 cluster, but not in the parvafox nucleus.

**Table 4:** List of genes locally enriched in the PV2 cluster of neurons

Genes	Complete name of the genes	Parvafox nucleus (Szabolcsi et al. 2017)	Parvafox nucleus (Girard et al. 2011)	Reticular thalamic nucleus (Allen brain atlas)
*Acat2*	Acetyl-coenzyme A acetyltransferase			
*Adcyap1r1*	Adenylate cyclase activating polypeptide 1 receptor 1			
*Adycap1*	Adenylate cyclase activating polypeptide 1	Yes	Yes (specific for parvafox)	
*AI852640*	Expressed sequence			
*Aldoc*	Aldolase C, fructose-biphosphate			
*Arl2*	ADP-ribosylation factor-like 2			
*Bag1*	BCL2-associated athanogene 1			
*Calb1*	Calbindin 1			
*Capn2*	Calpain 2			Yes
*Ccdc91*	Coiled-coil domain containing 91			
*Ccni*	Cyclin 1			Yes
*Cdh24*	Cadherin-like 24			
*Chat*	Choline acetyltransferase			
*Chn2*	Chimerin 2			
*Chrm2*	Cholinergic receptor, muscarinic 2, cardiac		Possible	
*Cox6c*	Cytochrome c oxidase			
*Crhr1*	Corticotropin releasing hormone receptor 1		Yes (restricted expression pattern)	Yes
*Cyb561*	Cytochrome b-561			
*Cygb*	Cytoglobin			Yes
*Cyp51*	Cytochrome p450 family 51			
*D830030K20Rik*	RIKEN cDNA			
*Dlk1*	Delta-like homolog (drosophila)			
*Efr3a*	EFR3 homolog A		Yes	Yes
*Emb*	Embigin			Yes
*Fdft1*	Farnesyl diphosphate farnesyl transferase			
*Fgfr1*	Fibroblast growht factor receptor 1			
*Gabra1*	GABA A receptor, subunit alpha 1			
*Gabrb2*	GABA A receptor subunit beta 2			
*Gabrq*	GABA A receptor			
*Gad1*	Glutamate decarboxylase 1		Negative in parvafox	Yes
*Gad2*	Glutamic acid decarboxylase			Yes
*Glra1*	Glycine receptor, alpha 1 subunit	Yes		
*Gm10413*	Predicted gene 10413			
*Gpc1*	Glypican 1			
*Gpr165*	G protein-couple receptor 165			
*Grm1*	Glutamate receptor, metabotropic 1			
*Hcn2*	Hyperpolarization-activated, cyclic nucleotide-gated ion channels		Yes	Yes
*Htr2c*	Serotonin receptor 2c			
*Inpp5j*	Inositol polyphosphate			Yes
*Insig1*	Insulin induced gene 1			
*Iqsec3*	IQ motif and Sec7 domain 3			Yes
*Iscu*	IscU iron-sulfur cluster scaffold homolog			
*Kcna1*	Potassium voltage-gated channel, shaker-related subfamily member 1		Yes	
*Kcna2*	Potassium voltage-gated channel, shaker-related subfamily member 2		Yes	Yes
*Kcnab2*	Potassium voltage-gated channel, shaker-related subfamily, beta member 2		Yes	
*Kcnab3*	Potassium voltage-gated channel, shaker-related subfamily, beta member 3	Yes	Yes	Yes
*Kcnc1*	Potassium voltage gated channel, shaw-related subfamily, member 1		Yes	Yes
*Kcnc3*	Potassium voltage gated channel, shaw-related subfamily, member 3			Yes
*Kcnk1*	Potassium channel, subfamily K		Yes	
*Lmo3*	LIM domain only 3			
*Lynx1*	Ly6/neurotoxin 1		Yes (lynx 2 specific for parvafox)	
*Nacc2*	Nucleus accumbens associated 2			
*Ndnf*	Neuron-derived neurotrophic factor			
*Nos1*	Nitric oxide synthase 1			
*Nova1*	Neuro-oncological ventral antigen 1			
*Nxph4*	Neurexophlin 4	Yes	Yes	
*Osbpl9*	Oxysterol binding protein-like 9			
*Parva*	Parvin, alpha			
*Penk*	Preproenkephalin	Yes	No	
*Pi4k2a*	Phosphatidylinositol 4-kinase type 2 alpha			
*Pnoc*	Prepronociceptin			Yes
*Ppm1a*	Protein phosphatase 1a			
*Pvalb*	Parvalbumin	Yes	Yes	Yes
*Rap1gap2*	RAP1 GTPase activating protein 2			
*Rab3b*	RAB3B, member RAS oncogene			
*Rgs10*	Regulator of G-protein signaling 10			
*S100a10*	S100calcium binding protein A10 (Calpactin)			
*Scn4b*	Sodium channel		Yes (restricted expression pattern)	Yes
*Serpini1*	Serine (or cysteine) peptidase inhibitor clade I. member 1		Yes	Yes
*Slc17a6*	Solute carrier family 17	Yes	Yes (specific for parvafox)	
*Slc25a*	Solute carrier family 25			
*Slc35g2*	Solute carrier family 35			
*Slc39a14*	Solute carrier family 39			Yes
*Slit1*	Slit homolog 1 (drosophila)			
*Snca*	Synuclein, alpha			
*Sncg*	Synuclein, gamma		No	Yes
*Socs5*	Suppressor of cytokine signaling			
*Sox2*	SRY (sex determining region Y)			
*Spp1*	Secreted phosphoprotein		Yes (restricted expression pattern)	Yes
*Steap2*	Six transmembrane epithelial antigen of prostate 2			
*Sv2c*	Synaptic vesicle glycoprotein 2c			
*Syt4*	Synaptotagmin IV			
*Tyr*	Tyrosinase			
*Vamp1*	Vesicle-associated membrane protein 1	Yes	Yes	Yes
*Vgf*	VGF nerve growth factor			

Gene expressed in the PV2 cluster. The other three columns indicate the expression of the same gene in other parvalbumin-positive structures, namely the hypothalamic parvafox-nucleus (Girard et al. 2011; Szabolcsi et al. 2017) and the reticular thalamic nucleus (Allen brain nucleotide-gated ion channels atlas)

**Fig. 10:**
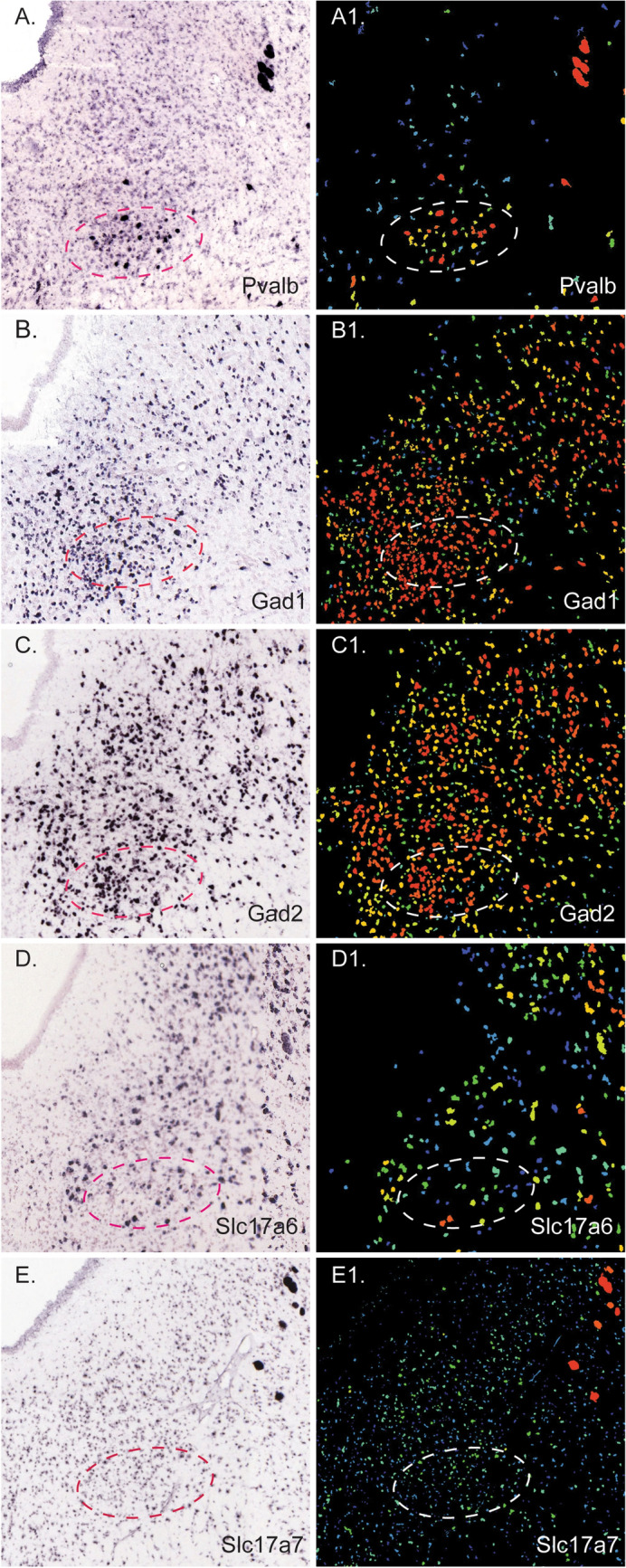
In situ hybridization (ISH) images of markers for GABA and glutamate at the level of the PV2 cluster. ISH images and the corresponding expression patterns of *Pvalb* (**A**, A1), the markers for GABA, namely *Gad1* (**B**, B1) and *Gad2* (**C**, C1) and the markers for glutamatergic neurotransmission, Slc17a6 (**D**, D1) and Slc17a7 (**E**, E1). In the expression images, the red color indicates strong expression, yellow indicates intermediate expression and green indicate low expression. The oval shows the approximate location of the PV2 cluster. All images are downloaded from the Allen Brain Atlas database (http://mouse.brain-map.org)

Contrary to the VGlut2-positive parvafox cells (Meszar et al. 2012; Girard et al. 2011), the region occupied by the PV2 cluster is rich in the expression of *Gad1* and *Gad2* (Fig. 10B, B1, C, C1), which is a strong indication that at least a proportion of PV2-neurons may use GABA as their neurotransmitter. Moreover, *Slc16a7* was faintly expressed and there were only rarely cells expressing Slc16a6 (respective markers for VGlut1 and VGlut2) (Fig. 10D, D1, E, E1).

## Discussion

The findings of this work show the PV2 cluster to be a cylindrical, cytoarchitectonic entity in the distalmost part of the PAG of the murine midbrain. Thanks to the expression of the calcium-binding protein Parv by its neurons, it can be distinguished from its surroundings. While it does overlay certain defined structures in and around the PAG, including the peritrochlear nucleus, the posterodorsal raphe nucleus and the laterodorsal tegmental nucleus (Franklin and Paxinos 2008), the PV2 cluster does not correspond to any of these structures in their entirety. The PV2 cluster was described as a novel entity being a main terminal field of axons arising in the parvafox nucleus of the lateral hypothalamus (Celio et al. 2013) and the LO/VLO region of the orbitofrontal cortex (Babalian et al. 2019). It is located ventrolateral to the aqueduct between the Bregma levels − 4.5 and − 4.96. In older atlas, this region was referred to as the *substantia grisea centralis, pars ventralis* (König and Klippel 1963) and most publications describing the divisions of the PAG fail to mention this region at all (Meller and Dennis 1986). Furthermore, the PV2 cluster does not correspond to any of the classical columns of the PAG described in the literature (Bandler and Shipley 1994; Shipley et al. 1991). The bilateral cluster contains on average of 475 cells per hemisphere and shows no sexual dimorphisms.

From a detailed study of gene expression in the Allen Brain atlas, we deduced that the genes *Gad1* and *Gad2*, typical of inhibitory GABA-neurons, are highly expressed in the region of the PV2 cluster. The genes *Slc17A6* and *Slc17A7*, encoding the vesicular glutamate transporters VGlut1 and VGlut2, on the other hand are rarely expressed in the region occupied by the PV2 cluster. We could indeed demonstrate that at least a certain percentage of Parv-positive neurons of the PV2 cluster express a VGAT-marker and the terminals are double stained with antibodies against GAD67. These neurons probably utilize GABA as a neurotransmitter, suggesting their inhibitory nature.

The tract-tracing studies on this hitherto undescribed PV2 cluster allowed to deduce its projections, dispersed throughout the entire brain, and include various structures involved in cardiovascular and respiratory control.

### Projections of the PV2 cluster of the PAG (Figs. 11, 12)

Various groups have studied the PAG and its projections extensively, but except for the work by the Aston-Jones group (injections in the supraoculomotor central gray and caudal ventrolateral PAG in rats), the injections rarely corresponded to the location of the PV2 cluster, but are located more rostrodorsally in the region of the Su3-nucleus (Henderson et al. 1998; Ennis et al. 1997; Van Bockstaele et al. 1991, 1989).

**Fig. 11:**
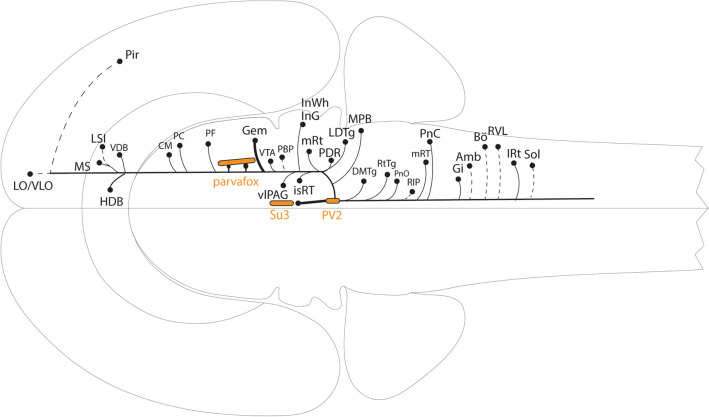
Schematic illustration after Swanson (Swanson 2004) of the efferent projections from the PV2 cluster. Horizontal view of the brain areas that receive projections from the PV2 cluster corresponding to Table 1. The density of the terminals is shown as solid (thicker), medium (thinner) or dashed (fainter) lines. The structures in orange represent the parvafox—Su3—PV2 circuitry. For abbreviations see Table 1

**Fig. 12:**
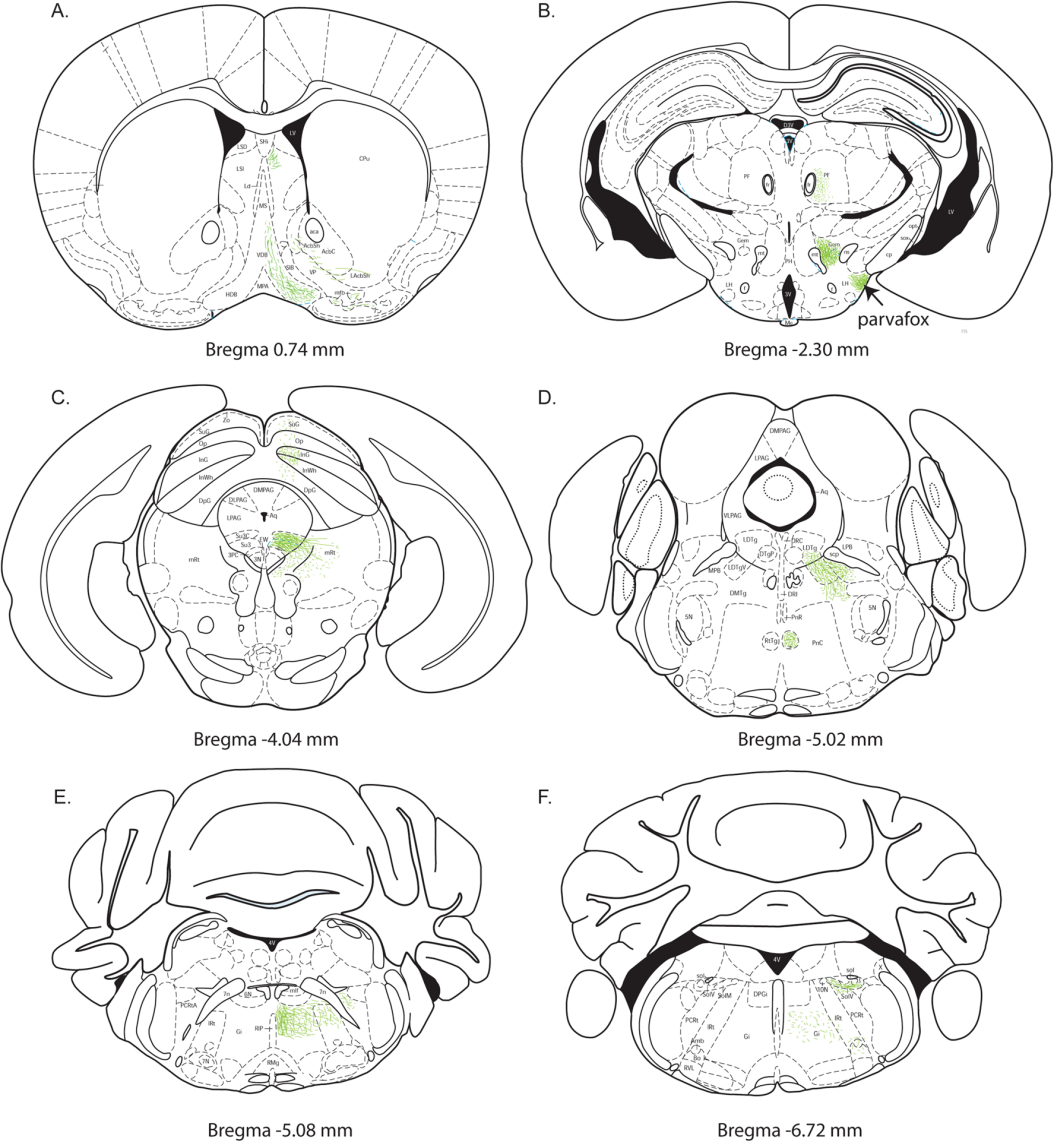
Drawings of the terminal endings of axons arising from the Parv expressing neurons of the PV2 cluster. Series of coronal drawings at 6 brain levels of the mouse brain (Franklin and Paxinos 2008), in which the projections of the PV2 cluster are represented. **A** The main projections to the septal regions and the diagonal band. **B** The projections to the parvafox nucleus and the gemini nuclei of the hypothalamus as well as the parafascicular nucleus. **C** The main projection to the Su3-nucleus as well as the projection to the superior colliculus. **D** The tegmental projections including the laterodorsal and reticulotegmental nuclei. **E** The projections within the gigantocellular nucleus and the intermediate reticular nucleus. **F** The hindbrain projections including the solitary nucleus, the gigantocellular nucleus, and the faint projections to the *Nucleus ambiguus* and Bötzinger complex. Drawing A: corresponds to level of Fig. 25 in (Franklin and Paxinos 2008); B: to Fig. 50; C: to Fig. 64; D: to Fig. 73; E: to Fig. 78; F: to Fig. 87

Injections at a Bregma level corresponding to that of the PV2 cluster in rats (− 6.65, − 8.60 (Celio et al. 2013)), but located slightly medial to the PV2 cluster, resulted in a diffuse pattern of labelled fibers distributed throughout the reticular formation, like our results. One branch of fibers terminated in the rostral medulla, including the RVLM, the LPGi and the *Nucleus ambiguus*. More caudally, terminals were remarked in the *Nucleus ambiguus* and the CVLM (Cameron et al. 1995). A further study, which included an injection that encompassed but was not restricted to the PV2 cluster, showed labelling predominantly in the ipsilateral CVLM (Chen and Aston-Jones 1996) (Figs. 11, 12).

Lesioning of a region of the caudal PAG of cats, corresponding to the location of the PV2 cluster in rats, caused a marked decrease in mean arterial pressure (MAP) in animals with an elevated MAP (Ward and Darlington 1987a), as well as a rapid decrease in arterial pressure and renal vascular resistance during slight hemorrhaging, during which the MAP would normally remain stable (Ward and Darlington 1987b).

### The PV2 cluster projects to cardiovascular sites

The PV2 cluster has a reciprocal relationship with the parvafox nucleus of the lateral hypothalamus: it is excited by its glutamatergic neurons and a subpopulation of PV2 neurons likely inhibits the parvafox with its neurotransmitter GABA. The function of the parvafox nucleus is yet unclear, but from its localization within the ventrolateral hypothalamus it is thought to be implicated in autonomic control. The parvafox nucleus harbors two cell populations, namely, a Parv-positive core and a shell of Foxb1-expressing neurons. The core projects mainly to the Su3 and the PV2 cluster of the PAG (Celio et al. 2013). The projections of the Foxb1-cells target both Su3- and PV2 cluster, but in addition attain various structures in the PAG like the lateral/dorsolateral parts and the adjacent cuneiform nucleus (Bilella et al. 2016).

Besides the reciprocal connection with the parvafox nucleus, the PV2 cluster densely innervates the Su3-nucleus of the PAG. The Su3 nucleus lies within the oculomotor complex between a NADPH-reactive cap (Carrive and Paxinos 1994) and a parvicellular band of cells, which projects mainly to the contralateral abducens nucleus (Van Bockstaele and Aston-Jones 1992). Even though the precise function of the Su3 nucleus has yet to be understood, it is known to project to the paragigantocellular nucleus of the medulla, specifically the rostral (RVLM) and caudal (CVLM) ventrolateral medulla, regions that are implicated in cardiovascular and respiratory control (Van Bockstaele et al. 1989, 1991; Chen and Aston-Jones 1996). The cardiovascular responses elicited by the RVLM are thought to be influenced by a direct projection from the PAG and by an indirect inhibitory projection via the CVLM (Chen and Aston-Jones 1996). Therefore, it is possible that the entire orbitofrontal cortex-parvafox-Su3-PV2 circuitry also has an indirect effect on these centers and the Su3 nucleus may modulate all the information from and to these structures.

The PV2 cluster may also directly influence other structures involved in cardiovascular and respiratory control through its projections to the *Nuclei ambiguus* and *solitarius*.

At the level of the trochlear nucleus and the dorsal raphe nucleus, labelled cells appear in the vlPAG, a region similar to that of the PV2 cluster after retrograde injections in the *Nucleus ambiguus* (Figs. 1E–F of (Ennis et al. 1997)). Anterograde injections into the same region of the PAG showed labelling in the *Nucleus ambiguus*, the periambigual region and the gigantocellular nucleus. Within the *Nucleus ambiguus*, the terminals intermingle with the cholinergic cells (Ennis et al. 1997), consistent with the findings shown in Fig. 7C. The periambigual region contains a majority of vagal preganglionic neurons projecting to the heart, which has been reported after injections of retrograde tracers in the pericardium (Bandler and Shipley 1994).

Since the PV2 cluster does not correspond to any of the classical columns, it is difficult to predict where the PV2 cluster precisely fits into this complex network of cardiovascular control. The results of these tracing studies show that the PV2 cluster is a further relay station within the PAG and may modulate cardiovascular and respiratory reactions according to the environment or emotional states via its partly inhibitory projection to the Su3-nucleus and the parvafox nucleus.

### Projections of the PV2 cluster to structures involved in respiration and vocalization

Various structures that receive projections from the PV2 cluster are involved in the central control of the respiratory system, namely, the Bötzinger complex, the retro- and periambigual areas and the ventral, ventrolateral and medial nuclei of the solitary tract.

Electrical stimulation of the caudal ventrolateral gray results in freezing and immobility, as well as hypotension (Depaulis et al. 1994; Keay et al. 1997b). In line with these observations, stimulation of this area in rats elicited a momentary increase in respiratory frequency, irregular breathing, followed by respiratory depression and no vocalization (Subramanian et al. 2008). This respiratory response corresponds to the freezing behavior observed.

The ipsilateral caudal ventrolateral medulla, a region which corresponds to the *Nucleus retroambiguus*, shows labelling after injections of Phaseolus vulgaris leucoagglutinin in the ventrolateral PAG (Chen and Aston-Jones 1996). In some of our cases, projections in this region were also observed, albeit only faintly. The *Nucleus retroambiguus* (sometimes referred to as the ventral respiratory nucleus (Felten 2016)), which also receives projections from the Foxb1-cells of the parvafox nucleus (Bilella et al. 2016), harbors premotor interneurons responsible for the production of the motor actions involved in vocalization (Roccaro-Waldmeyer et al. 2016; Subramanian and Holstege 2009). The interneurons within the *Nucleus retroambiguus* project to motor neurons within the spinal cord and supply accessory musculature associated with expiration (Felten 2016). Vocalization happens during a specific phase called post-inspiratory expiration, thus requiring an intimate relationship to the respiratory cycle. Interestingly, after lesioning of the parvafox nucleus, fewer 50 kHz ultrasonic vocalizations were observed during tickling (Roccaro-Waldmeyer et al. 2016), indicating the involvement of this region in the expression of a positive emotional state.

The *Nucleus tractus solitarii* (NTS) is a viscerosensory structure that receives information from respiratory, cardiovascular, gustatory, and gastrointestinal afferents. These afferents are topographically organized in various subnuclei in both the rostrocaudal and the mediolateral axes. There is some variation within the literature regarding these subdivisions, thus illustrating the complexity of the anatomy of this region. Afferent fibers from the respiratory system terminate in the commissural, intermedial and ventrolateral nuclei (Jänig 2006) or in the ventrolateral, ventral, medial, interstitial and commissural NTS (Kalia and Mesulam 1980; Loewy 1990). Additionally, while there is thorough proof of the topographical organization of the viscerosensory afferents to the NTS, there is no substantial evidence of such an organization of the second-order neurons of these pathways (Loewy 1990). Not only does the NTS project to other sensory relay centers, like the parabrachial nuclei (Loewy 1990), but it also sends projections to the areas harboring vagal premotor interneurons: the caudal, more lateral parts of the NTS innervate the caudal ventrolateral medulla, while the intermediate part of the NTS also innervates the *Nucleus ambiguus* (Ross et al. 1985).

The dorsal and ventral respiratory nuclei mutually inhibit each other. Additionally, the medial parabrachial nucleus acts as a respiratory pacemaker between these two and connections between the nuclei of the solitary tract and the medial parabrachial nucleus have been shown (Saper and Loewy 1980).

It is possible that the projections observed from the PV2 cluster to the ventrolateral, ventral, and medial subnuclei of the NTS, as well as to the medial parabrachial nucleus play an inhibitory role in the modulation of respiratory reactions.

While many of the direct respiratory reflexes (e.g., frequency response to hypercapnia) occurs directly within the brainstem via information received from peripheral chemo- and baroreceptors, higher brain centers must control the respiratory adjustments to the environment (visual, olfactory or acoustic information) or in emotionally driven states. Even though the PV2 cluster only projects faintly to areas harboring respiratory premotor neurons (e.g., the CVLM or the *Nucleus retroambiguus*), it does terminate extensively in other relay stations (e.g., NTS), therefore being capable of influencing these groups indirectly. Thus, the PV2 cluster presents a viable candidate as the main mediator, acting by integrating information stemming from the entire brain and adjusting respiration appropriately.

### Projections to the olfactory system

Both PV2 cluster and Su3 receive an input from the lateral orbitofrontal cortex but do not reciprocate this projection (Babalian et al. 2019).

In the hypothalamus, the parvafox nucleus and gemini nucleus receive dense projections from the PV2 cluster. The projections to the gemini nucleus appeared to extend in the VTA, located just caudal to it. Only few studies have focused on or mentioned the gemini nucleus, but within these it has frequently been mentioned in the context of the olfactory system. Cells from the polymorphic layer of the olfactory tubercle project directly to the region known as the gemini nuclei (Price et al. 1991).

### Projections to the gustatory system

The medial parabrachial nucleus remains a less-known brain region. In the 1970s, the autonomic connections between the brainstem and the hypothalamus were beginning to gain acceptance, but the precise connections remained elusive. In line with these studies, the parabrachial nuclei were found to be a relay station for visceral autonomic information. While the lateral parabrachial nucleus is thought to be responsible for non-gustatory visceral information mainly from the solitary tract, the neurons of the medial parabrachial nucleus are mainly taste-responsive (Saper and Loewy 1980; Saper et al. 2016). It remains to be seen whether the neurons that receive projections from the PV2 cluster are responsible for delivering gustatory or cardiovascular and respiratory information to the medial parabrachial nucleus.

### Projections to the visual system

The intermediate layers of the superior colliculus receive dense projections from the PV2 cluster. The superior colliculus plays a role in the orientation of the head and eye movements, as well as integrating visual information (e.g. the lower eye field registers the presence of a predator and activates the medial deep layers of the superior colliculus (Comoli et al. 2012)). The superior colliculus may receive an inhibitory input from the PV2 cluster and itself projects to the lateral PAG (Furigo et al. 2010), responsible for behavioral responses like hunting.

In conclusion, the PV2 cluster shows extensive projections mainly with cardiovascular and respiratory relay centers in the brain, but additionally projects to various structures involved in other sensory modalities. This highlights the possibility that the PV2 cluster is a mediator of autonomic control in response to environmental or emotional changes.

### The inhibitory effect of the PV2 cluster

The second main finding of our studies of the PV2 cluster is the probably inhibitory nature of a part of its neurons. Its terminals were positive for glutamic acid decarboxylase (GAD67), and the cell bodies expressed VGAT1, thus suggesting that at least a part of the cells of this Parv-positive cluster are GABAergic. The analysis of the gene expression provided further indications that the region occupied by the PV2 cluster is enriched in GABA-markers (Fig. 10). The expression of *Gad1* and *Gad2*, albeit not confined solely to the PV2 cluster, but rather heavily expressed throughout the caudal PAG, supplied us with further proof. Thus, while some neurons of the PV2 may use GABA as their neurotransmitter, it is possible that this pertains only to a fraction of the PV2 cluster. On the contrary, the expression of the markers for the vesicular glutamate transporters were rare (*Slc17a6*) or faint (*Slc17a7*).

Nevertheless, further *in-situ* hybridization or immunostaining studies will be needed to expand the knowledge about the neurotransmitters utilized by the neurons within the PV2 cluster.

An additional finding of our gene expression analysis was the presence of the receptor *Adcyap1r1* in the PV2 cluster. This is particularly interesting, as the parvafox nucleus strongly expresses the *Adcyap1* gene that encode a protein of the glucagon superfamily, namely PACA, which plays a role in growth and metabolism. Further functions that have been associated with PACA include the control of food intake, synaptic plasticity and antinociception. Given that the gene encoding the receptor is expressed in the region of the PV2 cluster, it is possible that the neurons of the parvafox nucleus use this peptide as a neuromodulator, which acts on its receptor in the PV2 cluster.

The PV2 cluster has now been described as its own entity for the first time. The results from this study suggest that parts of the neurons of the PV2 cluster may be inhibitory. How large this portion is and the neurotransmitter status of the other Parv-positive neurons in the PV2 cluster remains to be elucidated. Since the PV2 cluster has a reciprocal relationship with the parvafox nucleus of the lateral hypothalamus it is likely that this cluster of neurons is involved in a circuitry involving the orbitofrontal cortex, the parvafox nucleus, as well as the Su3- nucleus of the PAG.

### Limitations of the study

The mapping of the location of labelled neurons, axons, and terminals was not made in relation to Nissl-defined cytoarchitectonic boundaries. The boundaries, visually confirmed by three co-authors, were determined by comparing our images with those published in the mouse atlas (Franklin and Paxinos 2008). Despite the precision of the stereotaxic injections in the PV2 cluster and the use of various controls, it cannot be excluded that some adjacent Pvalb-Cre neurons were co-infected with the virus.

## Data Availability

Due to their storage in a university database, the datasets generated during the current study are not publicly available but are available from the corresponding author on reasonable request.

## References

[CR1] Alvarez-Bolado G, Celio MR (2016). The ventrolateral hypothalamic area and the parvafox nucleus: role in the expression of (positive) emotions?. J Comp Neurol.

[CR2] Babalian A, Eichenberger S, Bilella A, Szabolcsi V, Alvarez-Bolado G, Chun X, Celio MR (2019). The orbitofrontal cortex projects to the parvafoxnucleus of the ventrolateral hypothalamus and to its targets in the ventral periaqueductal gray matter in preparation. Brain Struct Funct.

[CR3] Bandler R, Carrive P (1988). Integrated defence reaction elicited by excitatory amino acid microinjection in the midbrain periaqueductal gray region of the unrestrained cat. Brain Res.

[CR4] Bandler R, Shipley MT (1994). Columnar organization in the midbrain periaqueductal gray: modules for emotional expression?. Trends Neurosci.

[CR5] Bandler R, Carrive P, Zhang MR (1991). Integration of somatic and autonomic reactions within the midbrain periaqueductal gray: viscerotopic, somatotopic and functional organization. Prog Brain Res.

[CR6] Bandler R, Keay KA, Floyd N, Price J (2000). Central circuits mediating patterned autonomic activity during active vs. passive emotional coping. Brain Res Bull.

[CR7] Behbehani MM (1995). Functional characteristics of the midbrain periaqueductal gray. Prog Neurobiol.

[CR8] Bilella A, Alvarez-Bolado G, Celio MR (2014). Coaxiality of Foxb1- and parvalbumin-expressing neurons in the lateral hypothalamic PV1-nucleus. Neurosci Lett.

[CR9] Bilella A, Alvarez-Bolado G, Celio MR (2016). The Foxb1-expressing neurons of the ventrolateral hypothalamic parvafox nucleus project to defensive circuits. J Comp Neurol.

[CR10] Burgdorf J, Wood PL, Kroes RA, Moskal JR, Panksepp J (2007). Neurobiology of 50-kHz ultrasonic vocalizations in rats: electrode mapping, lesion, and pharmacology studies. Behav Brain Res.

[CR11] Cameron AA, Khan IA, Westlund KN, Willis WD (1995). The efferent projections of the periaqueductal gray in the rat: a phaseolus vulgaris-leucoagglutinin study II descending projections. J Comp Neurol.

[CR12] Carrive P, Paxinos G (1994). The supraoculomotor cap: a region revealed by NADPH diaphorase histochemistry. NeuroReport.

[CR13] Carrive P, Dampney RA, Bandler R (1987). Excitation of neurones in a restricted portion of the midbrain periaqueductal gray elicits both behavioural and cardiovascular components of the defence reaction in the unanesthetised decerebrate cat. Neurosci Lett.

[CR14] Carrive P, Bandler R, Dampney RA (1989). Somatic and autonomic integration in the midbrain of the unanesthetized decerebrate cat: a distinctive pattern evoked by excitation of neurones in the subtentorial portion of the midbrain periaqueductal gray. Brain Res.

[CR15] Celio MR (1990). Calbindin D-28k and parvalbumin in the rat nervous system. Neuroscience.

[CR16] Celio MR, Babalian A, Ha QH, Eichenberger S, Clement L, Marti C, Saper CB (2013). Efferent connections of the parvalbumin-positive (PV1) nucleus in the lateral hypothalamus of rodents. J Comp Neurol.

[CR17] Chen S, Aston-Jones G (1996). Extensive projections from the midbrain periaqueductal gray to the caudal ventrolateral medulla: a retrograde and anterograde tracing study in the rat. Neuroscience.

[CR18] Cola RB, Babalian A, Alvarez-Bolado G, Celio MR (2020) Involvement of the lateral hypothalamic Parvafox-Foxb1 neurons in defensive behaviors. Abstract Theme F.2.e : Neuronal control of organ function. XII FENS Meeting, Glasgow 11–15 July

[CR19] Comoli E, Das Neves Favaro P, Vautrelle N, Leriche M, Overton PG, Redgrave P (2012). Segregated anatomical input to sub-regions of the rodent superior colliculus associated with approach and defense. Front Neuroanat.

[CR20] Davis FP, Eddy SR (2009). A tool for identification of genes expressed in patterns of interest using the allen brain atlas. Bioinformatics.

[CR21] Depaulis A, Keay KA, Bandler R (1992). Longitudinal neuronal organization of defensive reactions in the midbrain periaqueductal gray region of the rat. Exp Brain Res.

[CR22] Depaulis A, Keay KA, Bandler R (1994). Quiescence and hyporeactivity evoked by activation of cell bodies in the ventrolateral midbrain periaqueductal gray of the rat. Exp Brain Res.

[CR23] Depaulis A, Bandler R. (1991) The midbrain periaqueductal matter. Functional, anatomical and neurochemical organization, vol 213. NATO ASI Series, vol A. Plenum Press, New York and London

[CR24] Ennis M, Xu SJ, Rizvi TA (1997). Discrete subregions of the rat midbrain periaqueductal gray project to nucleus ambiguus and the periambigual region. Neuroscience.

[CR25] Felten DL. (2016) Motor systems: central control of respiration. In: Netter’s atlas of neuroscience (3rd Edition)

[CR26] Franklin KBJ, Paxinos G (2008). The mouse brain in stereotaxic coordinates.

[CR27] Furigo IC, de Oliveira WF, de Oliveira AR, Comoli E, Baldo MV, Mota-Ortiz SR, Canteras NS (2010). The role of the superior colliculus in predatory hunting. Neuroscience.

[CR28] Gerig AT, Celio MR (2007). The human lateral tuberal nucleus: immunohistochemical characterization and analogy to the rodent PV1-nucleus. Brain Res.

[CR29] Girard F, Meszar Z, Marti C, Davis FP, Celio M (2011). Gene expression analysis in the parvalbumin-immunoreactive PV1 nucleus of the mouse lateral hypothalamus. Eur J Neurosci.

[CR30] Henderson LA, Keay KA, Bandler R (1998). The ventrolateral periaqueductal gray projects to caudal brainstem depressor regions: a functional-anatomical and physiological study. Neuroscience.

[CR31] Hess WR (1935). Hypothalamus und die Zentren des autonomen Nervensystems: Physiologie.

[CR32] Hippenmeyer S, Vrieseling E, Sigrist M, Portmann T, Laengle C, Ladle DR, Arber S (2005). A developmental switch in the response of DRG neurons to ETS transcription factor signaling. PLoS Biol.

[CR33] Jänig W (2006) Nucleus tractus solitarii. In: Jänig W (ed) Integrative action of the autonomic nervous system. Cambridge University Press, pp 311–317

[CR34] Kalia M, Mesulam MM (1980). Brain stem projections of sensory and motor components of the vagus complex in the cat: II Laryngeal, tracheobronchial, pulmonary, cardiac, and gastrointestinal branches. J Comp Neurol.

[CR35] Keay KA, Crowfoot LJ, Floyd NS, Henderson LA, Christie MJ, Bandler R (1997). Cardiovascular effects of microinjections of opioid agonists into the ‘Depressor region’ of the ventrolateral periaqueductal gray region. Brain Res.

[CR500] König JFR, Klippel RA (1963). The rat brain.

[CR36] Keay KA, Crowfoot LJ, Floyd NS, Henderson LA, Christie MJ, Bandler R (1997). Cardiovascular effects of microinjections of opioid agonists into the ‘Depressor region’ of the ventrolateral periaqueductal gray region. Brain Res.

[CR37] Loewy AD, Loewy AD, Spyer KM (1990). Central autonomic pathways. Central regulation of autonomic functions.

[CR38] Meller ST, Dennis BJ (1986). Afferent projections to the periaqueductal gray in the rabbit. Neuroscience.

[CR39] Meszar Z, Girard F, Saper CB, Celio MR (2012). The lateral hypothalamic parvalbumin-immunoreactive (PV1) nucleus in rodents. J Comp Neurol.

[CR40] Panksepp J, Burgdorf J (2003). “Laughing” rats and the evolutionary antecedents of human joy?. Physiol Behav.

[CR41] Price JL, Slotnick BM, Revial MF (1991). Olfactory projections to the hypothalamus. J Comp Neurol.

[CR42] Roccaro-Waldmeyer DM, Babalian A, Muller A, Celio MR (2016). Reduction in 50-kHz call-numbers and suppression of tickling-associated positive affective behaviour after lesioning of the lateral hypothalamic parvafox nucleus in rats. Behav Brain Res.

[CR43] Ross CA, Ruggiero DA, Reis DJ (1985). Projections from the nucleus tractus solitarii to the rostral ventrolateral medulla. J Comp Neurol.

[CR44] Saper CB, Loewy AD (1980). Efferent connections of the parabrachial nucleus in the rat. Brain Res.

[CR45] Saper CB, Loewy AD, Swanson LW (2016). Commentary on: Saper CB, Loewy AD, Swanson LW, Cowan WM. (1976) direct hypothalamo-autonomic connections. brain research 117:305–312. Brain Res.

[CR46] Sessle BJ, Ball GJ, Lucier GE (1981). Suppressive influences from periaqueductal gray and nucleus raphe magnus on respiration and related reflex activities and on solitary tract neurons, and eVect of naloxone. Brain Res.

[CR47] Shipley MT, Ennis M, Rizvi TA, Behbehani MM, Depaulis A, Bandler R (1991). Topographicalspecificity of forebrain inputs to the midbrain periaqueductal gray: evidence for discrete longitudinally organized input columns. The midbrain periaqueductal gray matter.

[CR48] Siemian JN, Borja CB, Sarsfield S, Kisner A, Aponte Y (2019). Lateral hypothalamic fast-spiking parvalbumin neurons modulate nociception through connections in the periaqueductal gray area. Sci Rep.

[CR49] Spencer SE, Sawyer WB, Loewy AD (1989). Cardiovascular effects produced by L-glutamate stimulation of the lateral hypothalamic area. Am J Physiol.

[CR50] Subramanian HH, Holstege G (2009). The nucleus retroambiguus control of respiration. J Neurosci.

[CR51] Subramanian HH, Balnave RJ, Holstege G (2008). The midbrain periaqueductal gray control of respiration. J Neurosci.

[CR52] Swanson LW (2004). Brain maps: the structure of the rat brain.

[CR53] Szabolcsi V, Albisetti GW, Celio MR (2017). Parvalbumin-neurons of the ventrolateral hypothalamic parvafox nucleus receive a glycinergic input: a gene-microarray study. Front Mol Neurosci.

[CR54] Van Bockstaele EJ, Aston-Jones G (1992). Distinct populations of neurons in the ventromedial periaqueductal gray project to the rostral ventral medulla and abducens nucleus. Brain Res.

[CR55] Van Bockstaele EJ, Pieribone VA, Aston-Jones G (1989). Diverse afferents converge on the nucleus paragigantocellularis in the rat ventrolateral medulla: retrograde and anterograde tracing studies. J Comp Neurol.

[CR56] Van Bockstaele EJ, Aston-Jones G, Pieribone VA, Ennis M, Shipley MT (1991). Subregions of the periaqueductal gray topographically innervate the rostral ventral medulla in the rat. J Comp Neurol.

[CR57] Verberne AJ, Guyenet PG (1992). Midbrain central gray: influence on medullary sympathoexcitatory neurons and the baroreflex in rats. Am J Physiol.

[CR58] Verberne AJ, Struyker Boudier HA (1991). Midbrain central gray: regional haemodynamic control and excitatory amino acidergic mechanisms. Brain Res.

[CR59] Ward DG, Darlington DN (1987). A blood pressure lowering effect of lesions of the caudal periaqueductal gray: relationship to basal pressure. Brain Res.

[CR60] Ward DG, Darlington DN (1987). Lesions of the caudal periaqueductal gray prevent compensation of arterial pressure during hemorrhage. Brain Res.

[CR61] Yajima Y, Hayashi Y, Yoshii N (1980). The midbrain central gray substance as a highly sensitive neural structure for the production of ultrasonic vocalization in the rat. Brain Res.

[CR62] Zhang SP, Bandler R, Carrive P (1990). Flight and immobility evoked by excitatory amino acid microinjection within distinct parts of the subtentorial midbrain periaqueductal gray of the cat. Brain Res.

